# Analysing researchers’ outreach efforts and the association with publication metrics: A case study of Kudos

**DOI:** 10.1371/journal.pone.0183217

**Published:** 2017-08-17

**Authors:** Mojisola Erdt, Htet Htet Aung, Ashley Sara Aw, Charlie Rapple, Yin-Leng Theng

**Affiliations:** 1 Centre for HEalthy and Sustainable CitieS (CHESS), Wee Kim Wee School of Communication and Information, Nanyang Technological University, Singapore, Singapore; 2 Kudos Innovations Ltd., Oxfordshire, England; KU Leuven, BELGIUM

## Abstract

With the growth of scholarly collaboration networks and social communication platforms, members of the scholarly community are experimenting with their approach to disseminating research outputs, in an effort to increase their audience and outreach. However, from a researcher’s point of view, it is difficult to determine whether efforts to make work more visible are worthwhile (in terms of the association with publication metrics) and within that, difficult to assess which platform or network is most effective for sharing work and connecting to a wider audience. We undertook a case study of Kudos (https://www.growkudos.com), a web-based service that claims to help researchers increase the outreach of their publications, to examine the most effective tools for sharing publications online, and to investigate which actions are associated with improved metrics. We extracted a dataset from Kudos of 830,565 unique publications claimed by authors, for which 20,775 had actions taken to explain or share via Kudos, and for 4,867 of these full text download data from publishers was available. Findings show that researchers are most likely to share their work on Facebook, but links shared on Twitter are more likely to be clicked on. A Mann-Whitney U test revealed that a treatment group (publications having actions in Kudos) had a significantly higher median average of 149 full text downloads (23.1% more) per publication as compared to a control group (having no actions in Kudos) with a median average of 121 full text downloads per publication. These findings suggest that performing actions on publications, such as sharing, explaining, or enriching, could help to increase the number of full text downloads of a publication.

## Introduction

With the rapid development of new media technologies, more people are turning to online sources to seek information about science and scientific developments. Unlike traditional print and broadcast media, new web-based platforms have made it possible for audiences to step out of their passive roles and connect with the communicators themselves [1]. Hence, outside the scientific community, lay audiences are increasingly turning to other sources, besides mainstream journalistic ones, for scientific information [2]. They are also able to directly participate in scientific discussions by commenting on posts on online platforms and even producing their own content. The digital era has also given researchers the opportunity to manage the communication of their research outputs themselves. They can now reach a wider audience online via diverse channels and in different ways, thus a researcher’s scientific outreach is no longer restricted to the traditional academic community. Researchers are bringing attention to their publications on social media platforms like ResearchGate (https://www.researchgate.net) or Twitter (https://twitter.com) in an attempt to gain more readers, citations, and social impact [1].

Altmetrics can be described as new or alternative metrics based on activities on social media for measuring scholarly impact [3, 4]. However, in the literature, the term *altmetrics* is still very controversial, as it could also be seen as just a new form of *informetrics* [5], and the distinction between usage metrics and altmetrics is still unclear [6]. Many attempts have been made to define usage statistics. They could be described as an aggregation of the underlying usage data, for example the number of full text downloads or views of a publication [7]. Download statistics can be collected very quickly and provide an immediate measure of how many people have downloaded a publication. However, usage statistics could be a misleading measure of the impact of an article, as they are a simple but inaccurate measure of actual usage [8].

Traditional metrics such as citation counts or the h-index are slow indications of impact, for instance, citations could take years to appear. They also represent a narrow academic audience [9]. In contrast to traditional metrics, altmetrics reflect impact faster, as a tweet could appear within hours of a paper being published. In addition, altmetrics represent both academic and lay audiences [10]. Altmetrics however face some challenges. For example, the heterogeneous nature of altmetric sources [11] is a major challenge, as well as the inconsistency and lack of normalisation of altmetrics [10]. Also, very little is known about the motivation of users and the reasons why they mention and discuss research outputs on social media [3, 12]. Altmetrics are also very susceptible to manipulation [10]. For example, a study shows that automated Twitter bot accounts cause a lot of spam to scientific articles [13]. Download statistics are also vulnerable to manipulation and gaming, although many publishers have built in spam detection to prevent this [8]. A study shows that scholars have engaged in deceptive self-downloads on SSRN to lessen negative social comparisons with their peers [14]. Thus, to ensure data integrity, some strategies have been created, for example, the detection of irregular usage [15] and tracking suspicious activity by PLOS [16], as well as identifying patterns that indicate fraudulent downloads by SSRN [17]. Altmetrics also face many data quality issues [11] and there are no common standards across the different altmetric aggregators [18], though the publisher cooperative Crossref’s DOI Event Tracker pilot [19] may soon result in progress on that front. There have also been efforts made to recommend good practices like from the National Information Standards Organization (NISO) [20] and the European Expert Group on Altmetrics [21].

Since traditional metrics have been criticized for not comprehensively representing scholarly impact, not only researchers but publishers and service providers are starting to consider alternative tools to track the online presence of authors, articles and journals. Altmetric aggregators such as Altmetric.com (https://www.altmetric.com), Plum Analytics (http://plumanalytics.com), and Impactstory (https://impactstory.org) compile a range of altmetrics from different data sources and provide them to researchers to view and track [3, 4]. Some altmetrics are based on events such as views, reads, clicks, downloads, saves, bookmarks, tags, favourites, comments, reviews, links, shares, tweets, likes, or ratings [3]. Some also collect citations [4]. Alternative metrics have been corroborated as useful marketing tools by publishers in promoting articles and journals [22]. For example, Scopus (https://www.scopus.com) introduced “Altmetric for Scopus”, a third party web application that integrates into the sidebar of pages in Scopus, where researchers can track the mentions of different social media metrics and reference managers. Furthermore, editors and authors find it challenging to engage online with different audiences, and many readers find it difficult to locate and give feedback to publications, as these have been shared on different social media sites [23].

The above arguments and challenges motivate us to study the sources (i.e., online channels) that are commonly used to share publications, and to analyse the effectiveness of the sources for sharing a publication (i.e., to determine whether the number of links shared on online channels could be associated with an increase in the number of full text downloads) using Kudos (https://www.growkudos.com) as a case study in this paper. In the next section, we deliberate on the significance of new media in academia and discuss several features and services of social media platforms and altmetric services that provide support for researchers in their efforts to promote their research outputs and increase their outreach. We then look at related studies that investigate these systems. We present our reasons for choosing Kudos as a case study for this paper, we introduce the system and features provided to researchers. We then present the research questions we aim to address and the methodology used for the analysis. After this, the results of the analysis are presented. We then discuss our findings, limitations, and future work.

## Related work

The use of social media has been increasing rapidly over the past years. Although its rate of adoption has been slower in academia than in the general population [24], more scholars have started to see its usefulness, and some foresee that it may eventually influence tenure and promotion processes at academic institutions [25]. Scientists have acknowledged professional benefits to using social media tools, for example, a survey of highly cited US nano-scientists claim that the use of social media can contribute to a researcher’s scientific impact [26]. Scholars in the information science and technology field in North America are also starting to use social media tools professionally as they have been found to make research dissemination and connecting with peers more convenient [27].

A comprehensive literature review on the usage of social media in academia and in scholarly communication shows the interest and popularity amongst researchers in using social media platforms [28]. Social media are perceived as effective tools for the discovery and dissemination of research outputs [29]. A study by Nature shows that researchers use platforms like Google Scholar (https://scholar.google.com), LinkedIn (https://www.linkedin.com), ResearchGate, Academia.edu (https://www.academia.edu), Facebook (https://www.facebook.com), Mendeley (https://www.mendeley.com), and Twitter with varying degrees of usage intensity, and for diverse purposes, including maintaining their scholarly profiles, and sharing their research outputs [30].

Specifically, scholars have cited information dissemination as a major benefit of using Twitter [31]. In a study on the usage of Twitter by 587 scientists, it was reported that 32% claim to have posted an estimated number of 1001–5000 scientific tweets (tweets including scientific content or a science-related hash-tag), whereas 22% claim to have posted 101–500 scientific tweets, the majority aiming thereby to connect with fellow scientists [32]. Among digital humanities scholars, studies show that Twitter, functioning both as an information network and a social network, is seen as an important tool for informal communication [33]. Being mentioned on Twitter can help magnify the effect of interactions with journalists and those outside the scientific community [26]. Social impact measures based on tweets, such as the *twimpact factor*, have been proposed to complement traditional citation metrics [34].

Also, a survey of 2,414 researchers shows that researchers use YouTube (https://www.youtube.com), Slideshare (http://www.slideshare.net), and Flickr (https://www.flickr.com) to disseminate a wider range of research outputs, such as videos, presentation slides, or photos [29]. Meanwhile, in addition to traditional abstracts, some publishers such as PNAS, PLOS Biology, PLOS Genetics, PLOS Neglected Tropical Diseases, Frontiers in Ecology and the Environment, Behavioral Ecology, and Functional Ecology are making summaries available that are aimed at a wider audience [35]. This is seen as a move to make scientific research more visible and transparent. From a researcher's perspective, lay summaries provide them with a way to communicate their research to people outside their specialised field more easily [35]. For example, on Facebook, users are able to provide explanations and accompanying texts when they share links. The comments section also helps to encourage online discussion of an article. Making research more understandable to the general public could help to raise the awareness of the relevance of research outputs, and consequently enable faster adoption of practices [36].

There have been several studies investigating the effectiveness of systems that support researchers in disseminating their works and increasing their outreach. A recent study on Academia.edu attempted to measure the difference in citations between articles posted to Academia.edu and other articles from similar journals, using a sample of 31,216 papers [37]. Results from the study were however controversial as the methodology was found to be flawed and the findings possibly false [38].

In another study, the attributes of philosophy scholars on Academia.edu were examined [39]. Results showed that faculty tend to attract more profile views than students. A gender comparison also revealed that female philosophers did not attract more profile views than males. This suggests that academic capital drives philosophy uses of the site more than friendship and networking. A faculty advantage was also observed in law, history and computer science, except for females in law and females in computer science. Moreover, a correlation analysis found no correlation between traditional bibliometric measures and any Academia.edu metrics for philosophers [39].

In addition to Academia.edu, studies have also been conducted on other platforms such as ResearchGate, and Mendeley. A study on ResearchGate examined if the platform’s usage and publication data broadly reflect existing academic hierarchies and whether individual countries are set to benefit or lose out from the site. Findings indicate a moderate correlation between ResearchGate statistics and other rankings of academic institutions which suggests that ResearchGate use broadly reflects the traditional distribution of academic capital. Country-level analyses also found that Brazil, India, and some other countries seem to have a disproportionately large usage of ResearchGate. On the other hand, academics in China, South Korea, and Russia are making relatively little use of ResearchGate and may be missing opportunities offered by its use [40].

Mendeley readership counts have also been compared with citations for various social sciences and humanities disciplines. Results show that the overall correlation between Mendeley readership counts and citations for the social sciences was higher than for the humanities. There were also low and medium correlations between Mendeley bookmarks and citation counts in all the investigated disciplines, implying that they could be measuring different aspects of research impact [41]. A recent literature review [4] gives an overview of similar studies comparing altmetrics with citation, and a meta-analysis shows overall a low to medium correlation between most altmetrics and citation counts. Also, in several studies, altmetrics were compared with usage metrics and other altmetrics [4]. For example, in [42], a medium to high correlation was measured between ScienceDirect downloads and Mendeley readership counts, and also between ScienceDirect downloads and Scopus citations. Considerable differences were found in download statistics on the levels of discipline, journal, and document types [43]. Also, according to a study comparing downloads to citations in four disciplines (arts and humanities, computer science, economics, econometrics and finance, and oncology), 50–140 downloads could potentially correspond to one citation [44]. However, another study concludes from the log analysis of viewing full text articles, that scholars often download an article first and decide later about its relevance [45]. Also, publishers may not always be able to track all full text downloads of a publication as these might be available for download elsewhere [43]. Thus, it could be said that downloads are a statement of intent to use the publication [44], but this might not necessarily bring about any further use of the publication.

What however has not yet been extensively investigated is whether the outreach efforts of authors, (e.g., sharing their publications via social media and other online channels), could be associated with an increase in a publication’s metrics (e.g., the full text downloads of a publication). A study in the clinical pain sciences, albeit with a small sample of 16 PLOS ONE articles, does show that sharing via Facebook, Twitter, LinkedIn and ResearchBlogging.org is associated with an increase in the number of views and downloads of a publication, although the absolute effect size might not be considered substantial [46]. Thus, this motivates us to investigate how researchers share their work on social media and to try to measure how effective this is by analysing Kudos as a case study in this paper. Kudos is distinct from most of the services mentioned above as it enables researchers to manage multiple communication channels (rather than being an outreach channel in and of itself), and to track communications against a range of metrics (including citations, downloads and altmetrics) to understand their outreach efforts over time. Kudos achieves this by generating “trackable” links that researchers can share on social media as well as via other communications media such as email, or even offline communications (e.g. presentations, reading lists). Services like Twitter, Facebook and LinkedIn enable researchers to promote their research, but results are measured in terms of social media metrics such as views, shares and likes and not in terms of publication metrics such as downloads and citations; scholarly collaboration networks such as ResearchGate and Academia.edu are similar in that they enable promotion of work but with limited metrics, in these cases further restricted to other users within academia. Altmetric services such as Altmetric.com, Impactstory, and Plum Analytics track attention to research outputs across diverse data sources, but don’t show the full range of researchers’ own communication and dissemination efforts. Similarly, publisher websites and institutional repositories host content and provide a range of metrics—including both altmetrics and traditional metrics such as downloads and citations—but do not track researchers’ communications to determine where these may be improving results. Kudos combines data from all these sources, thereby building a dataset containing both communications data and metrics, and is therefore uniquely able to support analysis of the association between outreach efforts and metrics. Thus, due to these unique features and the data Kudos captures and cross-references, we select Kudos as a case study to investigate in this work.

## Case study: Kudos

Kudos (https://www.growkudos.com, launched in May 2014) is a web-based service that claims to help researchers, institutions and funders maximise the visibility and outreach of published articles. Kudos enables analysis not only of the current outreach of a publication, but also how that outreach has been achieved.

Kudos encourages researchers to take the following “actions”:

- Share: users can generate a link to their publications and share it online via Social Media, e.g., on Facebook, Twitter or LinkedIn, or they can also share the link through other channels e.g., email.
- Explain: users can provide a plain language explanation describing their work in a simpler manner.
- Enrich: users can upload links to additional resources, e.g., datasets or slides to supplement their publication.

**Fig 1:**
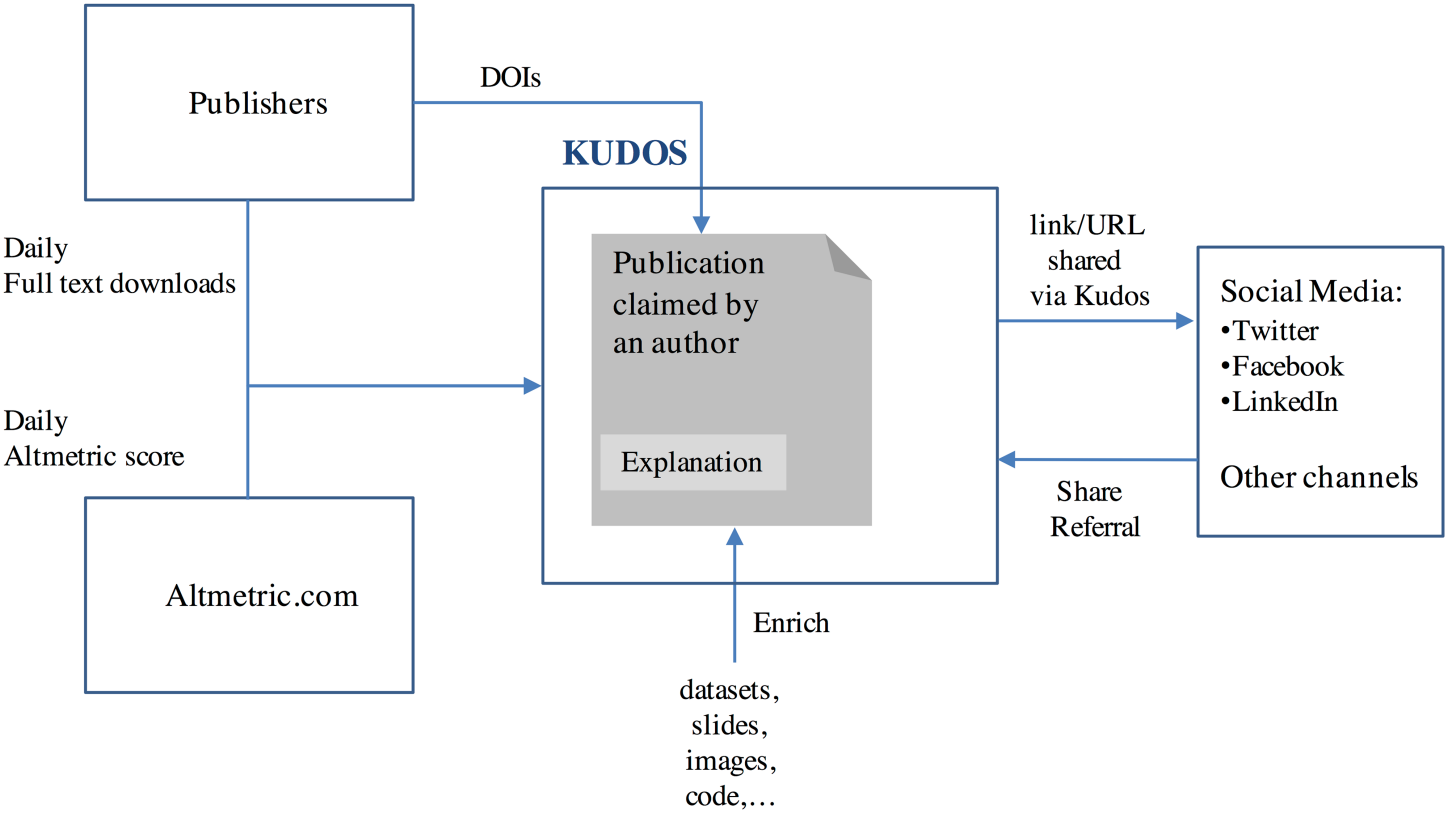
Overview of Kudos.

An overview of Kudos is shown in Fig 1, where a publication (essentially its DOI from its publisher) claimed by an author in Kudos can be explained and enriched with a dataset, slides, images or code. The author can also share a link to the publication by generating a share referral and sharing this via social media or other channels. Kudos collects daily full text downloads about this publication from its publisher, as well as the daily Altmetric Score from Altmetric.com.

Taking Kudos as a case study, we address the following research questions:

**RQ1**: Which online channels are most commonly used to share a publication on Kudos? Does the career level or discipline of the authors affect the choice of how publications are shared via Kudos?
**RQ2**: Which online channels are most effective for sharing a publication on Kudos? How do the numbers of links shared on online channels via Kudos correlate to an increase in the clicks on this link across different career levels and disciplines of the authors?
**RQ3**: Do any of the actions offered by Kudos have an association with the full text downloads of a publication?

## Methodology

In the methodology section, we present the data extraction process and metrics available for the analysis. We then explain the calculation of the increase in metrics which is used in the analysis. In addition, the methods used to recode career levels and disciplines are also presented in this section. We also present the data analysis methods applied to address each of the three research questions RQ1, RQ2, and RQ3 mentioned above.

### Data extraction and preparation

The dataset extracted from Kudos in February 2016 consisted of 830,565 unique publications which had been claimed by their authors in Kudos. Authors had undertaken Kudos “actions” for 22,170 of these: 4,610 had been shared and 20,775 had been explained. Of the 20,775 that had been explained, 4,499 had been further enriched.

Of the 22,170 publications in Kudos for which actions had been taken, 4,867 had full text download metrics (provided by publishers to Kudos), 1,485 had share referrals, and 10,521 had Altmetric Scores available. The metrics were available on a daily count from the time of claiming to the date of extraction from Kudos. Kudos tracks daily full text downloads of publications from participating publishers’ websites, and share referrals (clicks on the “trackable links” shared via Kudos to email, social media, etc.). Some publications were shared multiple times across the different channels, and sometimes multiple times via the same channel. When this occurred, the multiple records of a publication being shared were aggregated. Publication dates are not available in the metadata for all publications in Kudos. The dates on which publications were claimed by authors in Kudos ranged from 17 September 2013 to 08 January 2016. The datasets used for the analysis have been made available online [47].

### Metric calculation

**Fig 2:**
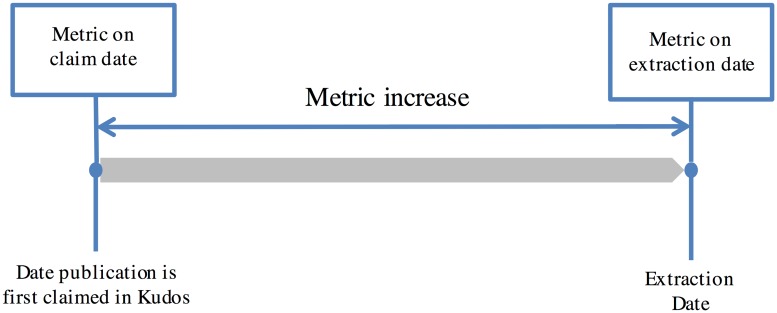
Metric increase calculation.

For our analysis, a metric increase was defined as an increase in a metric (i.e., full text downloads or share referrals) between the date the publication was first claimed in Kudos and the date of extraction from Kudos (30 January 2016) as shown in Fig 2. A single publication could be claimed by multiple users on different dates in Kudos, thus for such cases, the first available claim date is selected. If there was no change in the metric between the claim date and extraction date, the increase is taken as 0. If the claim date was the same as the extraction date, the increase is also taken as 0.

The assumption was made that the metrics are cumulative scores, thus the metric on the extraction date could only be greater or equal to the metric on the claim date. Unfortunately, the Altmetric Score from Altmetric.com does not respect this assumption as sometimes the Altmetric Score experienced a decrease in value due to changes in the calculation of the Altmetric Score by Altmetric.com. Furthermore, Kudos “trackable URLs” were only recognised by Altmetric.com following a change made part way through the time period represented in the dataset for this project. Thus, the Altmetric Score could not be considered further in the analysis.

### Recoding of career levels and disciplines

We recoded the different self-reported career levels and disciplines of the first authors to claim a publication into broader categories for the analysis. Career levels are grouped in five broad categories: professionals, students, researchers, faculty, and other career levels (See S9 Table). Regarding the discipline of the authors, we followed the OECD (Organisation for Economic Co-operation and Development, https://www.oecd.org/science/inno/38235147.pdf) classification scheme, which provides broader disciplines: natural sciences, engineering and technology, medical and health sciences, agricultural sciences, social sciences, humanities, and other disciplines (See S10 Table). “Aquatic Sciences” has been classified under “Natural Sciences” as most of its sub-disciplines belong to “Earth and related Environmental sciences”, according to the OECD classification. “Energy” has been placed in the category “Engineering and Technology”, as according to the OECD classification, “Energy and fuels” belongs to “Environmental engineering”. “Natural Sciences” has been expanded to encompass “Life Sciences” as they have many overlapping sub-disciplines, altnough “Life sciences” also has common sub-disciplines with “Medical and Health Sciences”. The OECD classification does not state “Life Sciences” as a category and due to the overlap with the other two categories, we decided against an independent category. “Innovation” has been placed in “Other” as it could be a part of all the categories.

### Methods

The data analysis methods applied to investigate the three research questions RQ1, RQ2, and RQ3 are presented below.

#### Data analysis method for RQ1

The aim of RQ1 is to investigate the most commonly used online channels to share a publication on Kudos. We thus consider only those publications in Kudos that have been shared on different social media channels, i.e., publications with share actions (n = 4,610). In this analysis, the self-reported career levels and disciplines of the first authors who claimed their publications on Kudos are considered. Data records on first authors not having a career level or a discipline were excluded from the analysis, consequently the total number of authors analysed per career level (n = 4,112) and discipline (n = 4,012) varies. A Chi-Square test is applied to determine statistical significance among the differences in career levels and disciplines.

#### Data analysis method for RQ2

RQ2 aims to investigate which online channels are most effective for sharing a publication. For this analysis, we investigate the self-reported career and discipline levels of the authors who claimed the publications in Kudos. We apply the Spearman correlation to determine the association between the number of links shared on the different social media channels and an increase in share referrals during the time between the claim date and extraction date. The Spearman correlation is applied since the underlying distribution is highly skewed. The analysis is performed on the 4,610 publications with share actions. Following [48]’s guidelines, the results are interpreted by the relationship strength as: *r* = .10 to .29 (small), *r* = .30 to .49 (medium), and *r* = .50 to 1.0 (large), regardless whether the values are positive or negative. The statistical significance level is shown at **p < .01 and *p < .05.

#### Data analysis method for RQ3

For RQ3, which aims to investigate if any of the actions offered by Kudos have an association with the full text downloads of a publication, two groups were compared with each other, a treatment group and a control group. An increase in metric (in full text downloads) was calculated in the same way as explained previously. An increase in full text downloads of a publication was measured between the first claim date in Kudos and the extraction date selected. In order to create two groups that are comparable, a data sampling procedure was performed. From the 830,565 unique publications claimed in Kudos, those having full text downloads available were extracted. This resulted in 71,081 publications with full text download information. The 71,081 publications were filtered into two parts: those with actions (4,867) and those without actions (66,214). For this analysis, the 4,867 publications with actions were taken to form the Treatment group. To form the Control group, 4,867 publications were randomly selected from the 66,214 publications without actions.

A correlation analysis was initially performed to measure the association between publications from the Treatment group (having actions) and an increase in full text downloads and the publications from the control group (having no actions) and an increase in full text downloads. The results did not offer much insight into the association between publications having actions or having no actions and an increase in full text downloads. We therefore looked into the characteristics of the publications considered for the analysis. We noticed that many of the publications in Kudos were only recently published, most between 2015 and 2011 (as can be seen in S3 Fig). Thus, the year of publication would need to be considered in the analysis. We also observed that many of the publications in Kudos were claimed in 2015 (as shown in S4 Fig), thus the time span between the claim date and the extraction date (30 January 2016) for most publications was limited, bringing about small increases in full text downloads. There were however also very large increases in full text downloads for a few publications (as can be seen in S12 Table). From this observation, for such a skewed distribution, the median would be the best choice of measure of central tendency. Thus, the median increase in full text downloads was compared between the Treatment group and the Control group by year. Publications having no information about their year of publication were excluded from the analysis. The Treatment group thus had n = 4,858 publications with publication years and the Control group n = 4,866 publications with publication years. In the analysis, a Mann-Whitney U Test was used to measure the difference between the medians of the groups. This test was chosen as it does not assume a normal distribution of the data and it is robust to outliers.

Furthermore, we also investigated the outliers that had shown a very high increase in full text downloads and realised this might be due to the type of publication. For example, a medical review having many authors had attracted an unusually large amount of full text downloads. We therefore analysed according to document type. We extracted document types from Scopus for the 4,858 publications of the Treatment group and the 4,866 publications of the control group having publication years. Document types from Scopus were available for 4,208 publications in the Treatment group, and for 4,155 in the Control group. We included only document types: *article*, *article in press*, and *conference paper* in the analysis. Other types of documents such as *review*, *editorial*, *erratum*, *letter*, *note* and *short survey* were excluded from the analysis as these could attract potentially different numbers of full text downloads compared to the more common article types [43]. Thus, for the analysis, we considered a total of n = 3,961 publications for the Treatment group and n = 3,851 for the Control group having document types article, article in press, or conference paper. See S11 Table for more details.

Additionally, we also performed an analysis on journal level, as this could also influence the downloads of a publication [43]. We merged journals with different parts but of the same subject area. For example, the Journal of Materials Chemistry A, B and C were merged into one journal, the Journal of Materials Chemistry. Also, the IUCr journals on Crystallography were merged to a single journal. The Treatment group had publications belonging to 245 different journals, the Control group had publications belonging to 292 different journals. However, only 11 journals had a sample size n > 20 in the Treatment group. As such, 1,132 publications (28.58%) from the Treatment group, and 2,325 publications (60.37%) from the Control group, could not be considered for analysis. A per year analysis per journal was not possible, as most of the journals did not have a sufficient sample size when further drilled down per year. The journals that had large enough sample sizes for analysis were mostly journals from the Royal Society of Chemistry, that allow a hybrid system of both gold open access for some publications, and green open access for the rest. We do not have the information on open access status for the publications in our dataset. As such, we are unable to conclusively rule out the open access advantage on full text downloads [49].

As disciplines could also have an effect on full text downloads [43], we also analysed by discipline level. As the subject area of the publications was not available in our dataset, the distribution of the self-reported disciplines of the first authors who claimed the publication were used (as shown in S5 and S6 Figs).

## Results and discussion

We present our findings of the analyses in this section, we first show the results of the descriptive analysis, and then we present the results of each research question.

### Descriptive findings

The total number of publications with actions in Kudos was 22,170. Of these, 4,610 (20.8%) had been shared, and 20,775 (93.7%) had been explained. Of those explained, 4,499 (21.7%) had been further enriched. Table 1 gives an overview of the metrics available for the publications having respective actions in Kudos. Of the 4,610 publications shared on Kudos, 9.8% have full text downloads and 32.2% had share referrals. Amongst publications with explain actions (in total 20,775), 22.8% have full text downloads, and 5.7% have share referrals. From the 4,499 publications with enrich actions, 67% have full text downloads, and 4.5% have share referrals.

**Table 1:** Publications with actions and their metrics.

	Publications with share actions (n = 4,610)	Publications with explain actions (n = 20,775)	Publications with enrich actions (n = 4,499)
**Full text downloads**	9.8%	22.8%	67.0%
**Share referrals**	32.2%	5.7%	4.5%

The percentages of metrics available (i.e., full text downloads and share referrals) for publications with share actions (n = 4,610), explain actions (n = 20,775), and enrich actions (n = 4,499) on Kudos.

### Findings for RQ1

**Fig 3:**
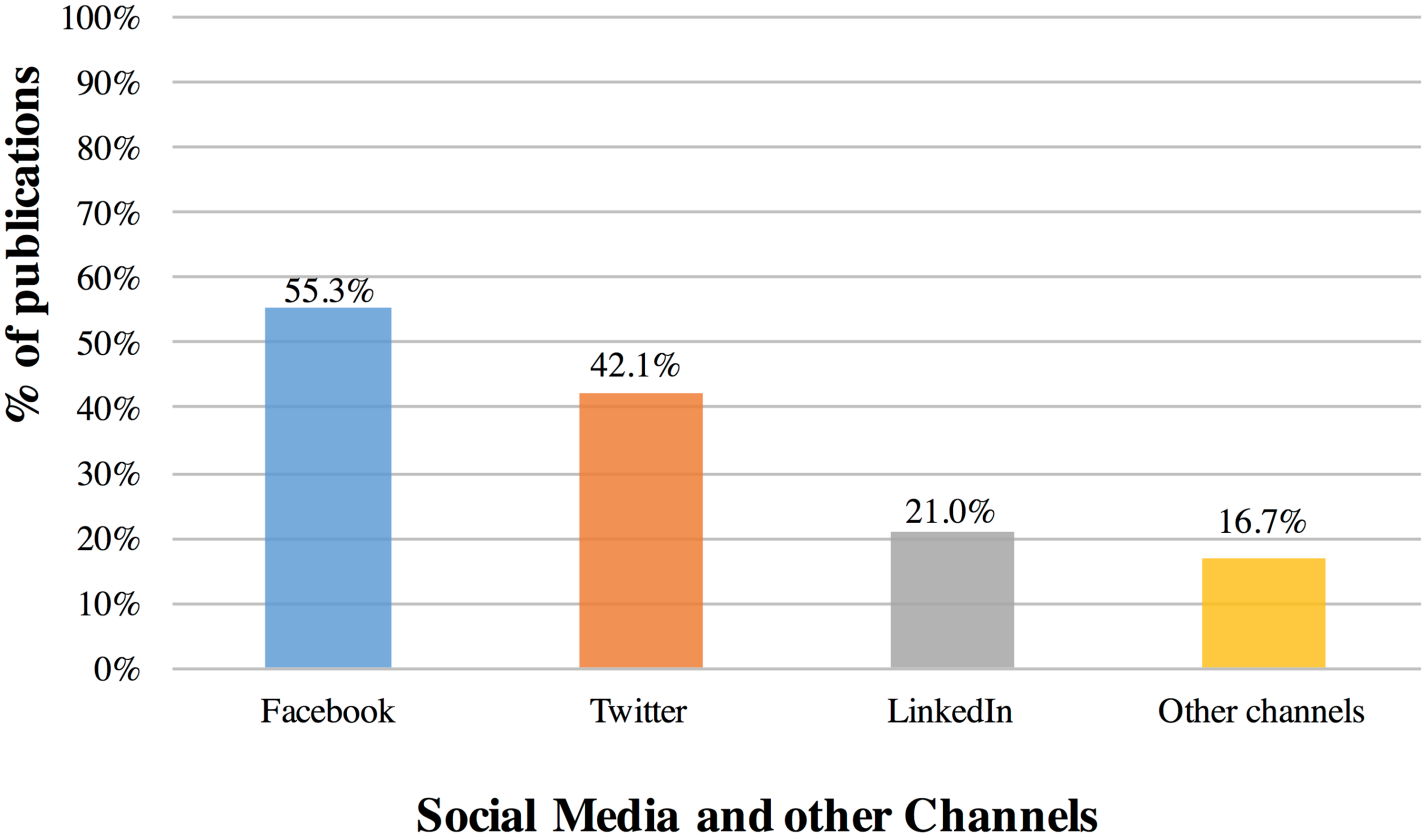
Distribution of shares via social media and other channels. Percentages of publications on Kudos with share actions (n = 4,610) that were shared via the three social media channels: Facebook (2,551), Twitter (1,941), and LinkedIn (969), as well as via other channels (772). Some publications were shared via multiple channels and some on different share dates. Multiple shares on the same channel on the same share date were counted only once in this analysis.

Fig 3 shows the percentage distributions of the different social media channels via which Kudos users shared their publications. Among the social media channels, Facebook is the most popular with 55.3%, followed by Twitter with 42.1%, then LinkedIn with 21.0%, and other channels (this includes sharing via email, other websites, networks, or offline) with 16.7%. However, LinkedIn was introduced by Kudos as an integrated sharing option later than Facebook and Twitter, in July 2015; when we only look at the 2,488 publications that had been shared after 07 July 2015, 62.3% had been shared via Facebook, followed by LinkedIn (38.9%), Twitter (35.2%), and other channels (4.5%). The distribution of shares on social media and other channels before and after 07 July 2015 are shown in S1 and S2 Figs.

**Fig 4:**
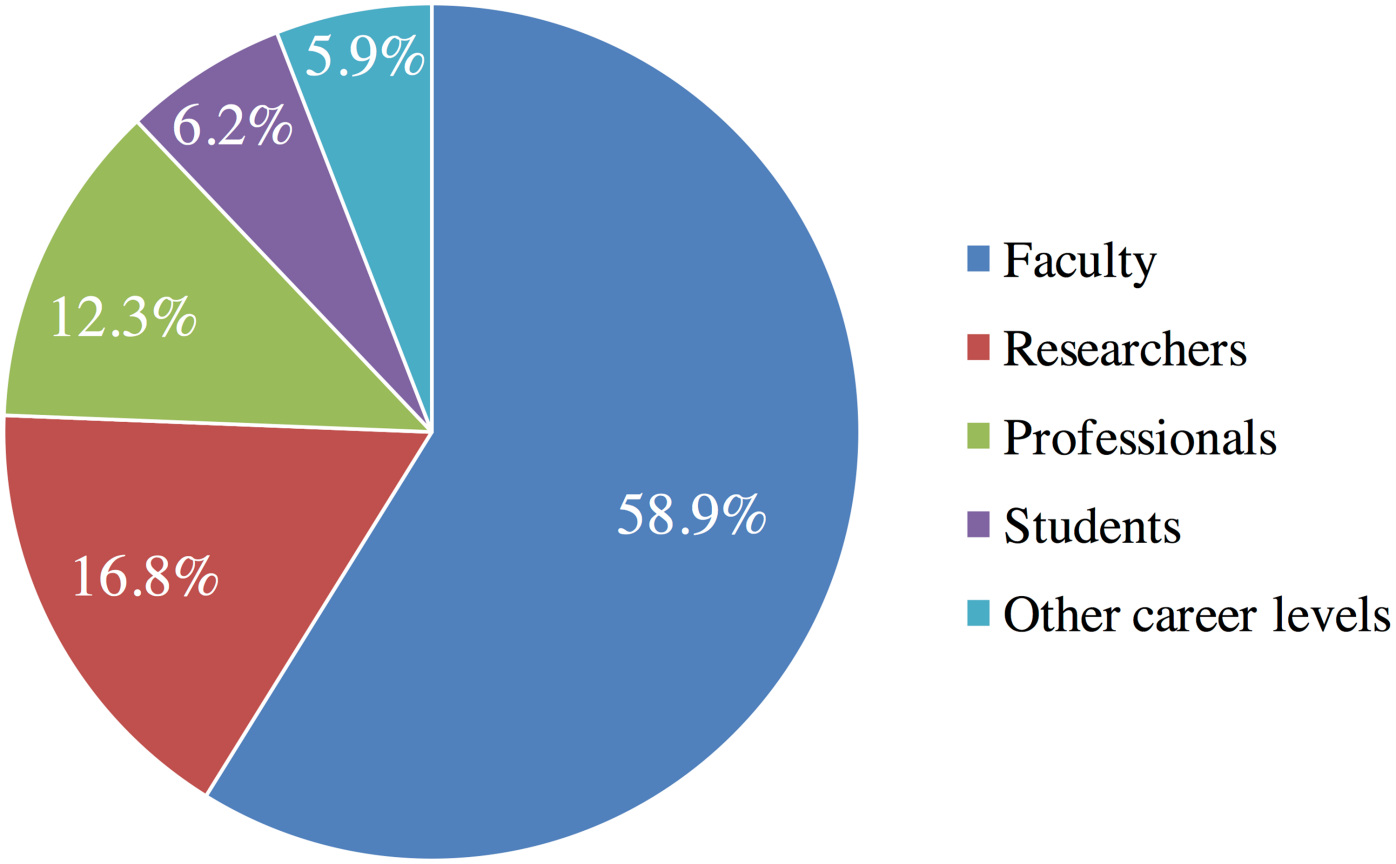
Distribution of career levels of authors. Percentage distributions of self-reported career levels of authors (n = 4,112), who first claimed and shared their publications via Kudos. Of the 4,112 authors, 2,420 were faculty, 689 researchers, 506 professionals, 256 students, and 241 did not specify their career levels or stated “other”.

**Fig 5:**
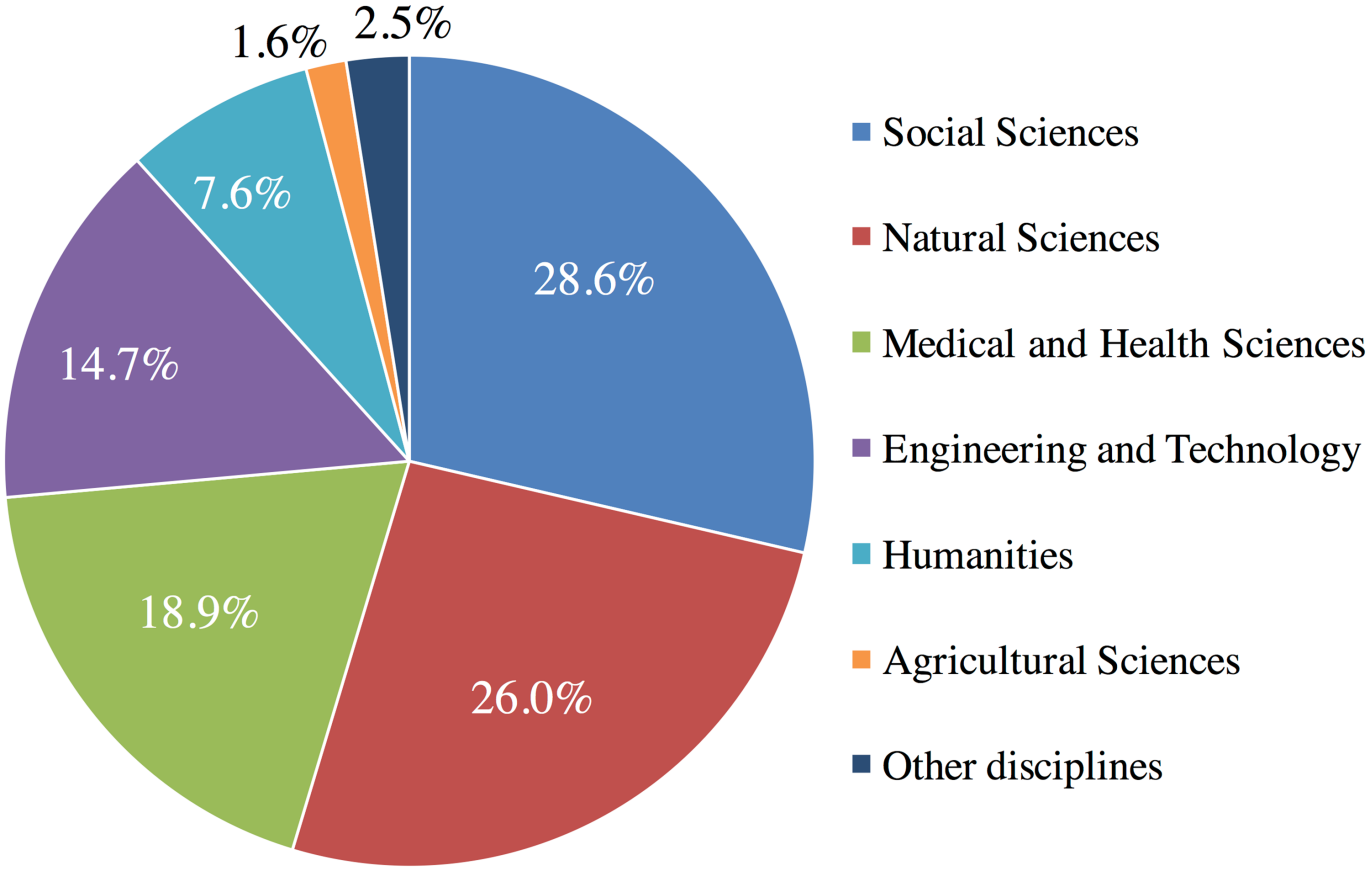
Distribution of disciplines of authors. Percentage distributions of self-reported disciplines of authors (n = 4,012), who first claimed and shared their publications via Kudos. 1,147 authors were from Social Sciences, 1,045 from Natural Sciences, 759 Medical and Health Sciences, 590 from Engineering and Technology, 304 Humanities, 65 Agricultural Sciences, and 102 from other disciplines.

As shown in Fig 4, among the 4,112 authors who first claimed and shared their publications on different social media channels in Kudos, the majority were faculty (58.9%), followed by researchers (16.8%), professionals (12.3%), and students (6.2%). In terms of discipline, of the 4,012 first authors who claimed and shared on social media channels (shown in Fig 5), 28.6% of authors were from Social Sciences, 26% from Natural Sciences, 18.9% from Medical and Health Sciences, and 14.7% from Engineering and Technology. Only 7.6% of authors from the Humanities, and 1.6% from Agricultural Sciences claimed and shared their publications in Kudos.

#### Career level differences in sharing on different social media channels

**Fig 6:**
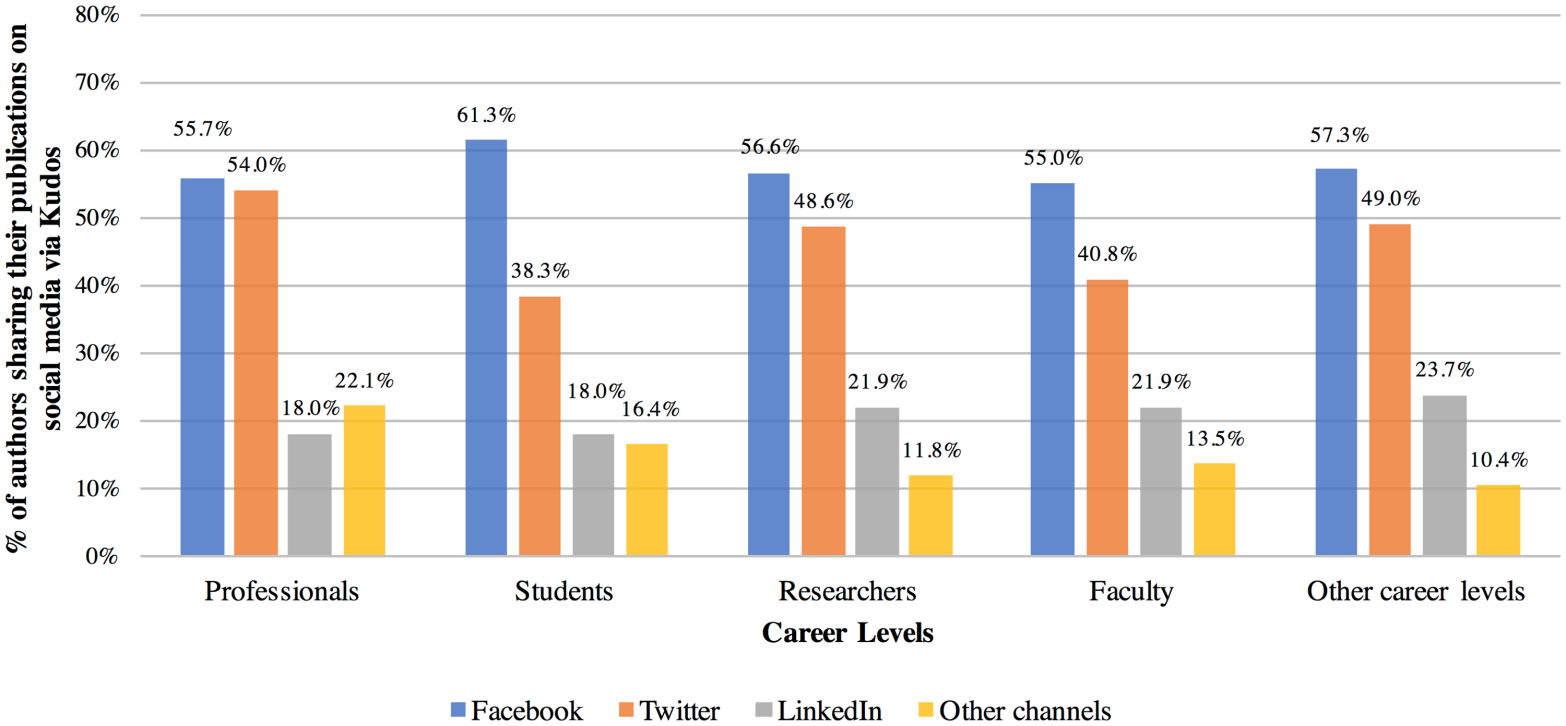
Distribution of authors, who shared their publications via social media, by career level. Professionals (n = 506), students (n = 256), researchers (n = 689), faculty (n = 2,420), and other career levels (n = 241) shared their publications via Kudos on Facebook, Twitter, LinkedIn, and other channels. Most authors shared their publications on multiple channels.

Fig 6 shows that around 60% of all career levels (professionals, students, researchers and faculty) shared their publications on Facebook, but, a Chi-Square test found no significant difference between the different career levels (p = .832, see S1 Table). Compared to students (38.3%) and faculty (40.8%), professionals (54.0%) and researchers (48.6%) used Twitter more as a sharing platform in Kudos, and this was statistically significant (p < .01, see S2 Table). In terms of sharing on LinkedIn, there was no significant difference among the different career levels (p = .170, see S3 Table). S4 Table shows the findings for “Other channels”.

#### Disciplinary differences in sharing on different social media channels

**Fig 7:**
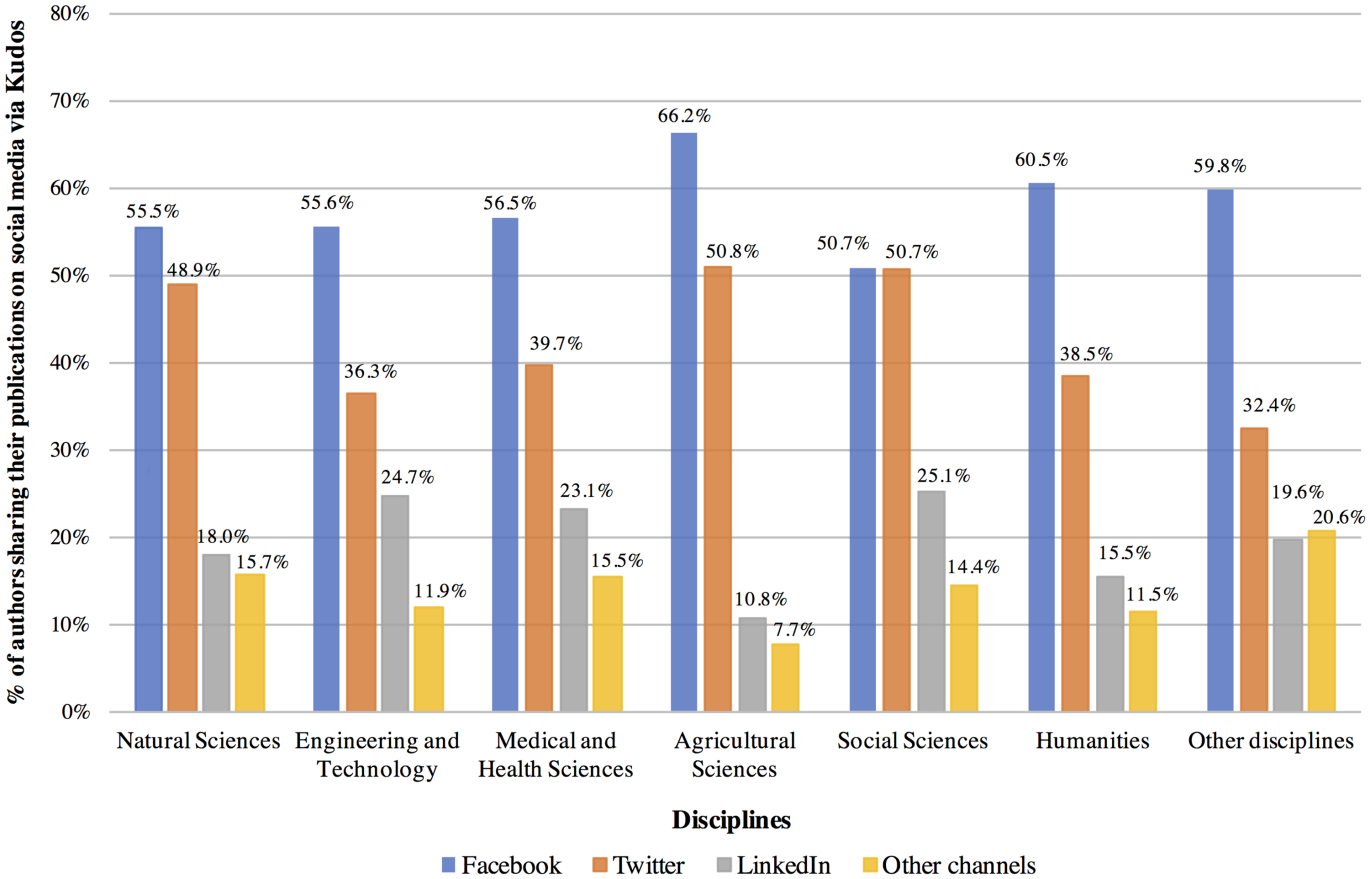
Distribution of authors, who shared their publications via social media, by discipline. Authors, who shared their publications via Facebook, Twitter, LinkedIn, and other channels were from Social Sciences (1,147), Natural Sciences (1,045), Medical and Health Sciences (759), Engineering and Technology (590), Humanities (304), Agricultural Sciences (65), and other disciplines (102). Most authors shared their publications on multiple channels.

As seen in Fig 7, overall, more than 50% of the authors across all disciplines shared their publications on Facebook. In particular, authors from Agricultural Sciences and Humanities shared their publications the most on Facebook (66.2%, and 60.5% respectively). Significant differences among the disciplines were found (p < .01, see S5 Table). The distribution varied for using Twitter as a sharing platform among disciplines, from 36.3% for Engineering and Technology to 50.8% for Agricultural Sciences and 50.7% for Social Sciences. This disciplinary difference is significant at p < .01 (See S6 Table). More than 10% of authors from all disciplines shared their publications via LinkedIn and this was found to be statistically significant (p < .01, see S7 Table), with 25.1% from Social Sciences and 24.7% from Engineering and Technology. S8 Table shows the findings for the group “Other channels”. Interestingly, 34.7% more of authors from Engineering and Technology, and 36.4% more of authors from Humanities shared their publications on Facebook than on Twitter.

### Findings for RQ2

The findings in Table 2 show that there are small but statistically significant Spearman correlations between the number of links shared on the three social media channels and an increase in share referrals. Sharing on Twitter shows a small correlation (rho = .28, *p* < .01), followed by LinkedIn (rho = .12, *p* < .01), and Facebook (rho = .11, *p* < .01). The results are based on n = 1,485 shared links that had been clicked.

**Table 2:** Spearman correlations between shares on social media and an increase in share referrals.

	Shares on Facebook	Shares on Twitter	Shares on LinkedIn	Shares on other channels
**Increase in Share Referrals**	.11**	.28**	.12**	-.00

Spearman correlations are calculated between the number of shares made on Facebook, Twitter, LinkedIn, and other online channels, and an increase in share referrals (n = 1,485).

*p < .05 (2 tailed).

**p < .01 (2 tailed).

The correlations between the number of shares on social media channels and an increase in share referrals by career level and discipline are also investigated. As shown in Table 3, on all the three social media channels, small but statistically significant correlations are found between the number of shares made by researchers and an increase in share referrals. For students, results show a medium statistically significant correlation for Facebook (rho = .32, *p* < .01). For faculty, a small correlation is found for LinkedIn (rho = .12, *p* < .01), and a medium correlation for Twitter (rho = .32, *p* < .01). A small correlation is also found between the number of shares made by professionals and an increase in share referrals on Twitter (rho = .27, *p* < .01).

**Table 3:** Spearman correlations between shares on social media and an increase in share referrals by career level.

	Shares on Facebook	Shares on Twitter	Shares on LinkedIn	Shares on other channels
**Professionals**	.11	.27**	.04	.02
**Students**	.32**	.15	.03	.18
**Researchers**	.15*	.17*	.17*	.06
**Faculty**	.04	.32**	.12**	-.03
**Other career levels**	.16	.38**	.16	-.22*

Spearman correlations are calculated for each of the career levels. The correlations are calculated between the number of shares made on Facebook, Twitter, LinkedIn, and other online channels, and an increase in share referrals by career level. Career levels comprised professionals (n = 506), students (n = 256), researchers (n = 689), faculty (n = 2,420), and other career levels (n = 241).

*p < .05 (2 tailed).

**p < .01 (2 tailed).

Small to large Spearman correlations between the number of shares made on Twitter and an increase in share referrals are seen across all disciplines in Table 4. The correlation results (ranging from small to medium significant correlations for all channels) show that the more links shared by Medical and Health Sciences authors, the more likely they are to be clicked on.

**Table 4:** Spearman correlations between shares on social media and an increase in share referrals by discipline.

	Shares on Facebook	Shares on Twitter	Shares on LinkedIn	Shares on other channels
**Natural Sciences**	.00	.33**	-.01	-.07
**Engineering and Technology**	-.03	.28**	.30**	-.03
**Medical and Health Sciences**	.23**	.35**	.21**	.23**
**Agricultural Sciences**	.40	.49*	.33	.12
**Social Sciences**	.11*	.14**	.17**	-.04
**Humanities**	.19*	.29**	.05	-.18*
**Other disciplines**	.15	.53**	.14	-.08

Spearman correlations are calculated for each of the disciplines. The correlations are calculated between the number of shares made on Facebook, Twitter, LinkedIn, and other online channels, and an increase in share referrals by discipline. The disciplines comprised Social Sciences (1,147), Natural Sciences (1,045), Medical and Health Sciences (759), Engineering and Technology (590), Humanities (304), Agricultural Sciences (65), and other disciplines (102).

*p < .05 (2 tailed).

**p < .01 (2 tailed).

### Findings for RQ3

Overall for all publications, the Treatment group (n = 4,858) had a significantly higher median average of 149 increase in full text downloads (23.1% more) per publication in comparison to the median average of 121 of the Control group (n = 4,866). The Mann-Whitney U value was found to be significant at (U = 10669164, Z = -8.31, *p* < .001). The results were not remarkably affected by filtering by document type. When considering only document types: article, article in press, and conference paper, the Treatment group (n = 3,961) still had a significantly higher median average of 158 increase in full text downloads (23.4% more) per publication as compared to the Control group (n = 3,851) with a median average of 128 increase in full text downloads per publication (U = 6929816, Z = -7.00, *p* < .001). The results are shown in Table 5.

**Table 5:** Mann-Whitney U test for different document types.

	Median		U	Z	*p*
	Treatment group	Control group			
**All document types**	149.00 (n = 4,858)	121.00 (n = 4,866)	10669164	-8.31	<.001
**Only document types: article, article in press, conference paper**	158.00 (n = 3,961)	128.00 (n = 3,851)	6929816	-7.00	<.001

Mann-Whitney U Tests are calculated between the Treatment group (publications having actions) and the Control group (publications having no actions) for all document types, as well as for only the document types: article, article in press, and conference paper.

For the following analyses, we only consider the document types: article, article in press, and conference paper. We drilled down to investigate an increase in full text downloads for different publication years, different journals, and different disciplines of the first authors who claimed the publications. The results for the different publication years are shown in Table 6. The results show that for almost all years, the Treatment group had higher median averages than the Control group. In particular, this difference is significantly higher in 2014 (U = 252948, Z = -4.64, *p* < .001), in 2012 (U = 191595, Z = -6.17, *p* < .001), and in 2011 (U = 76438, Z = -6.21, *p* < .001). 2016 and the other earlier years (including 2008) had too small sample sizes of n < 40 for the Treatment group (see S3 Fig), thus these comparisons need to be interpreted with caution. Some years could not be considered in the analysis at all, as such, not all years are presented here.

**Table 6:** Mann-Whitney U test for different publication years.

	Median		U	Z	*p*
	Treatment group	Control group			
**2015**	152.00 (n = 478)	137.00 (n = 699)	158114	-1.56	.118
**2014**	377.50 (n = 602)	263.50 (n = 976)	252948	-4.64	<.001
**2013**	168.00 (n = 611)	188.00 (n = 635)	182661	-1.79	.074
**2012**	126.00 (n = 1,404)	89.00 (n = 347)	191595	-6.17	<.001
**2011**	136.00 (n = 784)	89.50 (n = 262)	76438	-6.21	<.001
**2010**	212.00 (n = 27*)	100.00 (n = 165)	1894	-1.25	.213
**2009**	112.00 (n = 11*)	83.00 (n = 147)	613	-1.34	.181
**2008**	203.50 (n = 4*)	67.00 (n = 86)	109	-1.23	.217
**2007**	99.00 (n = 11*)	66.00 (n = 72)	320	-1.03	.304

Mann-Whitney U Tests are calculated between the Treatment Group (publications having actions) and the Control Group (publications having no actions) for the years 2015–2007. Due to small sample sizes, not all years in the dataset could be considered in the analysis.

* Due to small sample sizes (n < 40), results should be interpreted with caution.

The results for individual journals are shown in Table 7. Journals that had too small sample sizes of n < 40 for the Treatment group, were not considered in the analysis. The Treatment group showed a significant higher increase in full text downloads than the Control group for 7 of the journals: Analyst, Chemical Communications, CrystEngComm, Dalton Transactions, Journal of Materials Chemistry, Physical Chemistry Chemical Physics, and RSC Advances. However, several of the journals presented in Table 7 (including CrystEngComm and Dalton Transactions), have rather small sample sizes for the Treatment group and thus the results should be interpreted with care.

**Table 7:** Mann-Whitney U Test for different journals.

	Median		U	Z	*p*
	Treatment group	Control group			
**Analyst**	213.00 (n = 1,437)	149.50 (n = 50)	26593	-3.13	.002
**Analytical Methods**	80.00 (n = 993)	107.00 (n = 50)	16251	-4.13	<.001
**Chemical Communications**	397.00 (n = 73)	180.00 (n = 300)	6526	-5.36	<.001
**CrystEngComm**	214.00 (n = 25*)	88.50 (n = 84)	533	-3.73	<.001
**Dalton Transactions**	395.00 (n = 23*)	124.00 (n = 173)	913	-4.21	<.001
**IUCrJ**	137.00 (n = 21*)	161.50 (n = 20*)	210	-0.01	.990
**Journal of Materials Chemistry**	254.00 (n = 46)	126.50 (n = 272)	3400	-4.95	<.001
**Nanoscale**	233.00 (n = 37*)	215.00 (n = 61)	891	-1.74	.082
**Physical Chemistry Chemical Physics**	343.00 (n = 54)	151.00 (n = 200)	2971	-5.07	<.001
**RSC Advances**	141.00 (n = 93)	106.50 (n = 268)	9783	-3.09	.002
**Science of the Total Environment**	415.00 (n = 27*)	261.50 (n = 48)	418	-2.54	.011

Mann-Whitney U Tests are calculated between the Treatment Group (publications having actions) and the Control Group (publications having no actions) for 11 journals: Analyst, Chemical Communications, CrystEngComm, Dalton Transactions, Journal of Materials Chemistry, Physical Chemistry Chemical Physics, and RSC Advances. Due to small sample sizes, not all journals in the dataset could be considered in the analysis.

* Due to small sample sizes (n < 40), results should be interpreted with caution.

The results of the analysis of the increase in full text downloads by disciplines are presented in Table 8. The Treatment group showed a significant higher increase in full text downloads than the Control group for the disciplines Natural Sciences, Engineering and Technology, and Medical and Health Sciences. The sample sizes for some of the disciplines were rather small (n<40).

**Table 8:** Mann-Whitney U test for different disciplines.

	Median		U	Z	*p*
	Treatment group	Control group			
**Natural Sciences**	202.00 (n = 1,034)	123.00 (n = 2,178)	923597	-8.25	<.001
**Engineering and Technology**	187.00 (n = 313)	122.00 (n = 678)	85538	-4.91	<.001
**Medical and Health Sciences**	262.00 (n = 185)	136.00 (n = 455)	34002	-3.81	<.001
**Agricultural Sciences**	156.00 (n = 23*)	109.50 (n = 44)	449	-0.75	.452
**Social Sciences**	45.00 (n = 87)	89.00 (n = 102)	3918	-1.40	.161
**Humanities**	0.00 (n = 14*)	0.00 (n = 15*)	99	-0.33	.740
**Other disciplines**	73.50 (n = 36*)	155.00 (n = 49)	587	-2.63	.009

Mann-Whitney U Tests are calculated between the Treatment Group (publications having actions) and the Control Group (publications having no actions) for 6 disciplines: Natural Sciences, Engineering and Technology, and Medical and Health Sciences, Agricultural Sciences, Social Sciences, and Humanities, and other disciplines.

* Due to small sample sizes (n < 40), results should be interpreted with caution.

### Discussion of findings

In this study, we investigated three research questions. RQ1 aimed to investigate the most commonly used channels for users on Kudos to share their publications. Results show that Facebook was the most commonly used platform, followed by Twitter and LinkedIn. This same trend can be seen across all career levels and disciplines. More than half of the users shared their publications on Facebook across all career levels and disciplines. Most users however shared their publications on multiple channels, with more than 30% sharing their publications on at least two channels. Furthermore, the dataset was however slightly biased towards faculty, with more than half of the authors (58.9%, n = 2,420) being faculty. Also, findings for the discipline Agricultural Sciences must be interpreted with care, as the sample sizes were small for sharing on Twitter and LinkedIn with n < 40.

RQ2 aimed to investigate how effective researchers on Kudos were in sharing their publications on social media. Overall, the results show that sharing a Kudos generated URL on any of the popular social media channels (Facebook, Twitter, or LinkedIn) is associated with an increase in the clicks on this link (share referrals). Although Facebook might be the most commonly used platform, it seems that links shared on Twitter are more likely to be clicked on across all disciplines, and almost all career levels (except for students). In addition, researchers in the fields of medical and health sciences were moderately effective in sharing their research across all social media channels. This could be a motivation for the scholarly community to share their publications more on social media. The results are in line with other studies such as [50], which argue that most faculty are aware of different social media sites and agree that they play an important role in the academic field. We however cannot exclude any effects due to self-clicks, clicks from co-authors, or any other deceptive behaviour.

RQ3 aimed to analyse if any actions offered by Kudos had an association with the increase in full text downloads of a publication. Our study does not aim to generally compare the full text downloads of publications, but rather to analyse an increase in full text downloads for a publication from the time the author first claimed it in Kudos up till the data extraction date for this study. The aim is to analyse whether publications in Kudos having actions performed on them have a higher increase in full text downloads in this period when compared to publications in Kudos without any actions performed on them. The calculation of an increase in full text downloads in such a period is thus dependent on the publication having been claimed in Kudos and Kudos having tracked the full text downloads over this period. Thus, we are unable to expand this analysis to include papers that have not been claimed by authors in Kudos in the Control group as neither the increase in full text downloads can be calculated for them, nor does the period in Kudos for the analysis exist in such a case (there would be no claim date).

Several different data analysis approaches were tested on the dataset, but they did not yield conclusive results. For instance, individual actions were analysed using a correlation test to examine the association between actions and an increase in full text downloads, and between actions and an increase in share referrals. It was considered if a publication had an action or not, but the number of times the action was performed on the publication was not considered for the analysis. The increase in metric was calculated in the same way as previously mentioned. The findings however did not show that any specific actions could be associated with an increase in any of the metrics, and as such these analyses are not presented here.

The results of Mann-Whitney U tests show that overall the Treatment group had a significantly higher median average than the Control group, independent of document type. This finding was partially supported when analysed per publication year and journal. However, since most publication years and journals had too small sample sizes, the results have to be interpreted cautiously. Moreover, as most publications in the Treatment group and Control group had been published in journals belonging to the Royal Society of Chemistry, this means the Treatment group and Control group are rather homogeneous in discipline, with most publications coming from the broader field of chemistry. The Treatment group however had a large number of publications published in the journal the “Analyst”. But still in an attempt to drill down by discipline, we used the self-reported disciplines of the first author who claimed the publication in Kudos. S5 and S6 Figs show that about 60% of these authors said they belong to Natural Sciences (which includes Chemistry as a subject). Also, many authors did not specify their discipline, especially in the Treatment group. Thus the findings cannot be generalised to all disciplines. Overall, however, the findings do suggest the benefit of performing actions, such as sharing, explaining, or enriching publications which might help to increase the visibility and outreach of scientific works in new media landscapes, but neither the magnitude of impact nor the scope of outreach could be determined. In future, with more data collected, the analysis might become more insightful.

It is however important to note that social behaviours and expectations change in reaction to social statistics, rankings and public evaluation [51]. As scholars pay more attention to numbers, they also change their behaviours to influence their standing or rank. One reaction could be managing their online appearances and making strategic choices and efforts to improve ranking factors by employing gaming strategies to manipulate the numbers, with the aim of maximizing their metrics or rank [51]. For example, even established scholars are known to engage in deceptive self-downloads to improve the number of downloads when compared to their peers [14]. Gaming is considered illegitimate, and a misrepresentation of one’s impact, it breeds distrust and is thus mostly done in secrecy. In contrast, a more positive reaction would be to improve the characteristics that are being measured [51]. In the case of altmetrics, this would be to strive to publish high quality research outputs and to strive to make these known to a wide audience on social media. Furthermore, it should also be clarified that in this work, we have at most analysed the effectiveness of sharing on social media channels and how this improves the outreach of research publications, but we do not claim to have investigated any relation to dissemination or research impact as defined in [46].

### Limitations

There are some limitations to this study. This study by no means aims to generalize the findings made here to the entire research community. The dataset extracted from Kudos represents only a small fraction of researchers and published papers worldwide. The profile information about authors who have claimed publications in Kudos is self-reported, and we only analyse authors who claimed and shared in Kudos, thus, some results might be biased and the results cannot be generalized to all researchers and faculty. A number of authors and publications had to be excluded from the analysis due to insufficient data, such as missing dates of publication, or missing career level or discipline information for authors.

Moreover, this dataset could be criticised as being biased and represent only a narrow group of researchers as the services offered by Kudos might potentially attract a certain profile of users (e.g., already active on social media and aware of how to promote their publications). This can only be investigated with a user survey of Kudos’ users, which is out of scope for this study. However, it should be noted, that the services offered by Kudos are not restricted to any specific group of users, nor has any group of users been specifically targeted as potential users. Kudos is freely accessible to all who wish to use its services and we have had no influence over the users of Kudos, nor the publications they claim in the system. We also have had no influence over the actions they took on Kudos.

Furthermore, the treatment group could be criticised as having a potential selection bias as the users of Kudos might have chosen their most promising papers (which could already have had some downloads or attracted some attention on social media) to further perform actions on in Kudos to boost these papers’ metrics. This can also only be resolved by investigating the motivations of the users of Kudos in a user study.

Due to the limited sample size of the Treatment group (3,961 publications), the possible variations of analyses were limited. When broken down into smaller samples for example, per year, per journal, per discipline, most of the sample sizes got too small for analysis. We could also not compare publications on a per month basis as most publications did not have this information available. Also, due to the small sample sizes, drilling down per month led to even smaller sample sizes, in most cases too small for a comparison.

Some characteristics of the publications and authors were not considered in this study, such as geographical location of the author, open access status of the publication, and daily citation counts for publications. Thus there remain unanswered questions such as, does the geographical location of the author affect the sharing of publications in Kudos? Do open access publications receive more actions and full text downloads? Do actions in Kudos correlate to increased citation counts? Further work could be undertaken to explore other sources for metadata and metrics to overcome limitations in data availability.

## Conclusion

In this paper, we investigate the most effective actions that researchers could take, and channels that they could use to communicate their research outputs, in terms of the increase in full text downloads of the publications. The results show that Facebook is the most popular channel, but that links shared on Twitter are more likely to be clicked on. Moreover, we also find that actions performed in Kudos, such as sharing via social media and other channels, are associated with 23.1% more full text downloads. Citation counts were not analysed in this study so its findings do not claim that an increased rate of full text downloads is associated with a higher citation count in future.

We believe the findings from this study will be useful for individuals and institutions to improve and update understanding of researchers’ outreach efforts and the potential increase in a publication’s metrics. The findings may encourage researchers to use communications media more strategically for building the readership of their work. The findings could also motivate improved provision by publishers and service providers of metadata, metrics and services to support further analysis.

In the future, it would be interesting to gain more understanding of the scholarly community’s strategies of promoting their research, their opinion of disseminating research outputs on social media, and how they keep track of their research outputs. We could also study the information sharing behaviour, habits, and experiences of users on different social media channels, considering their demographics, such as the age group of the authors, career level, discipline, geographical location, etc. Furthermore, sharing via other channels such as wikis, blogs, forums, etc., could also be studied further.

## Supporting information

S1 Fig Distribution of shares via social media and other channels, before 07 July 2015. Percentages of publications on Kudos with share actions (n = 2,218) that were shared via Facebook (1,042), and Twitter (1,119), as well as via other channels (661), before 07 July 2015. LinkedIn was introduced by Kudos as an integrated sharing option in July 2015. Some publications were shared via multiple channels and on different share dates. Multiple shares on the same channel on the same share date were counted only once in this analysis.
(TIFF)

S2 Fig Distribution of shares via social media and other channels, after 07 July 2015. Percentages of publications on Kudos with share actions (n = 2,488) that were shared via the three social media channels: Facebook (1,551), Twitter (877), and LinkedIn (969), as well as via other channels (111), after 07 July 2015. LinkedIn was introduced by Kudos as an integrated sharing option in July 2015. Some publications were shared via multiple channels and on different share dates. Multiple shares on the same channel on the same share date were counted only once in this analysis.
(TIFF)

S3 Fig Distribution of publication years. The publication years for the publications with publication dates available, and restricted to publications with document types: article, article in press, and conference paper for the Treatment group (n = 3,961) and the Control group (n = 3,851).
(TIFF)

S4 Fig Distribution of claim dates. The month and year the publications were first claimed by an author in Kudos for publications with publication dates available, and restricted to publications with document types: article, article in press, and conference paper for the Treatment Group (n = 3,961) and the Control Group (n = 3,851).
(TIFF)

S5 Fig Disciplines in Treatment Group. Percentage distribution of disciplines of the first authors who claimed the publications in Kudos. For publications with publication dates available and restricted to publications with document types: article, article in press, and conference paper in the Treatment Group (n = 1,692): Natural Sciences (1,034), Engineering and Technology (313), Medical and Health Sciences (185), Agricultural Sciences (23), Social Sciences (87), Humanities (14), and other disciplines (36).
(TIFF)

S6 Fig Disciplines in Control Group. Percentage distribution of disciplines of the first authors who claimed the publications in Kudos. For publications with publication dates available and restricted to publications with document types: article, article in press, and conference paper in the Control Group (n = 3,521).: Natural Sciences (2,178), Engineering and Technology (678), Medical and Health Sciences (455), Agricultural Sciences (44), Social Sciences (102), Humanities (15), and other disciplines (49).
(TIFF)

S1 Table A Chi-Square test for sharing on Facebook across career levels. For career levels: professionals (n = 506), students (n = 256), researchers (n = 689), faculty (n = 2,420), and other career levels (n = 241) who shared their publications via Kudos on Facebook.
(PDF)

S2 Table A Chi-Square test for sharing on Twitter across career levels. For career levels: professionals (n = 506), students (n = 256), researchers (n = 689), faculty (n = 2,420), and other career levels (n = 241) who shared their publications via Kudos on Twitter.
(PDF)

S3 Table A Chi-Square test for sharing on LinkedIn across career levels. For career levels: professionals (n = 506), students (n = 256), researchers (n = 689), faculty (n = 2,420), and other career levels (n = 241) who shared their publications via Kudos on LinkedIn.
(PDF)

S4 Table A Chi-Square test for sharing on other channels across career levels. For career levels: professionals (n = 506), students (n = 256), researchers (n = 689), faculty (n = 2,420), and other career levels (n = 241) who shared their publications via Kudos on other channels. * Due to small sample sizes (n < 40), results should be interpreted with caution.
(PDF)

S5 Table A Chi-Square test for sharing on Facebook across different disciplines. Authors from Social Sciences (1,147), Natural Sciences (1,045), Medical and Health Sciences (759), Engineering and Technology (590), Humanities (304), Agricultural Sciences (65), and other disciplines (102), who shared their publications via Kudos on Facebook. * Due to small sample sizes (n < 40), results should be interpreted with caution.
(PDF)

S6 Table A Chi-Square test for sharing on Twitter across different disciplines. Authors from Social Sciences (1,147), Natural Sciences (1,045), Medical and Health Sciences (759), Engineering and Technology (590), Humanities (304), Agricultural Sciences (65), and other disciplines (102), who shared their publications via Kudos on Twitter. * Due to small sample sizes (n < 40), results should be interpreted with caution.
(PDF)

S7 Table A Chi-Square test for sharing on LinkedIn across different disciplines. Authors from Social Sciences (1,147), Natural Sciences (1,045), Medical and Health Sciences (759), Engineering and Technology (590), Humanities (304), Agricultural Sciences (65), and other disciplines (102), who shared their publications via Kudos on LinkedIn. * Due to small sample sizes (n < 40), results should be interpreted with caution.
(PDF)

S8 Table A Chi-Square test for sharing on other channels across different disciplines. Authors from Social Sciences (1,147), Natural Sciences (1,045), Medical and Health Sciences (759), Engineering and Technology (590), Humanities (304), Agricultural Sciences (65), and other disciplines (102), who shared their publications via Kudos on other channels. * Due to small sample sizes (n < 40), results should be interpreted with caution.
(PDF)

S9 Table Re-coding of career levels. The career levels of the first authors who claimed publications on Kudos were re-coded using the five broad OECD (Organisation for Economic Co-operation and Development, https://www.oecd.org/science/inno/38235147.pdf) career categories: Professionals, students, researchers, faculty, and other career levels.
(PDF)

S10 Table Re-coding of disciplines. The disciplines of the first authors who claimed publications in Kudos were recoded using the OECD (Organisation for Economic Co-operation and Development, https://www.oecd.org/science/inno/38235147.pdf) classification scheme, which provides seven broad disciplines: Natural Sciences, Engineering and Technology, Medical and Health Sciences, Agricultural Sciences, Social Sciences, Humanities, and other disciplines.
(PDF)

S11 Table Scopus document type. The document type was retrieved from Scopus for the publications in the Treatment group (n = 4,867) and the Control group (n = 4,867). Only 4,208 had document types available in the Treatment group, and only 4,155 had document types available in the Control group. The document types: article, article in press, conference paper, editorial, erratum, letter, note, review, and short survey were available.
(PDF)

S12 Table Full Text downloads for publications in treatment group and control group. Full text downloads for publications on the day they were first claimed by an author in Kudos, on the extraction date from Kudos for this study (30 January 2016), and the calculated increase in full text downloads between the claim date and extraction date, for publications with publication dates available and restricted to publications with document types: article, article in press, and conference paper for the Treatment group (n = 3,961) and the Control group (n = 3,851).
(PDF)
