# Supplementary figures and images for: Analysing researchers’ outreach efforts and the association with publication metrics: A case study of Kudos
Source: PLoS One. 2017 Aug 17;12(8):e0183217. doi: 10.1371/journal.pone.0183217 (PMC5560533; doi:10.1371/journal.pone.0183217)

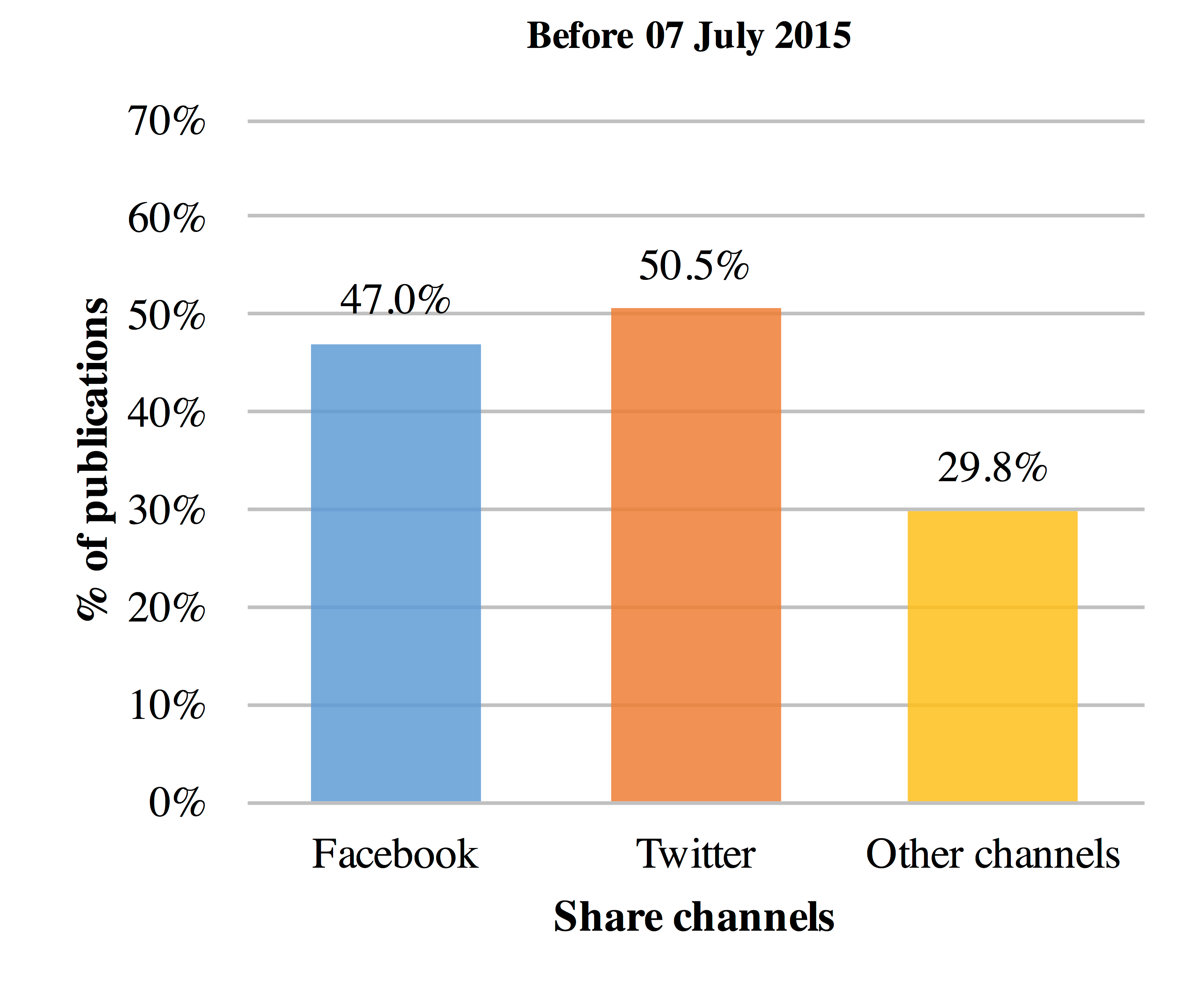

Supplement: S1 Fig — Percentages of publications on Kudos with share actions (n = 2,218) that were shared via Facebook (1,042), and Twitter (1,119), as well as via other channels (661), before 07 July 2015. LinkedIn was introduced by Kudos as an integrated sharing option in July 2015. Some publications were shared via multiple channels and on different share dates. Multiple shares on the same channel on the same share date were counted only once in this analysis. (TIFF) [file pone.0183217.s001.tiff]

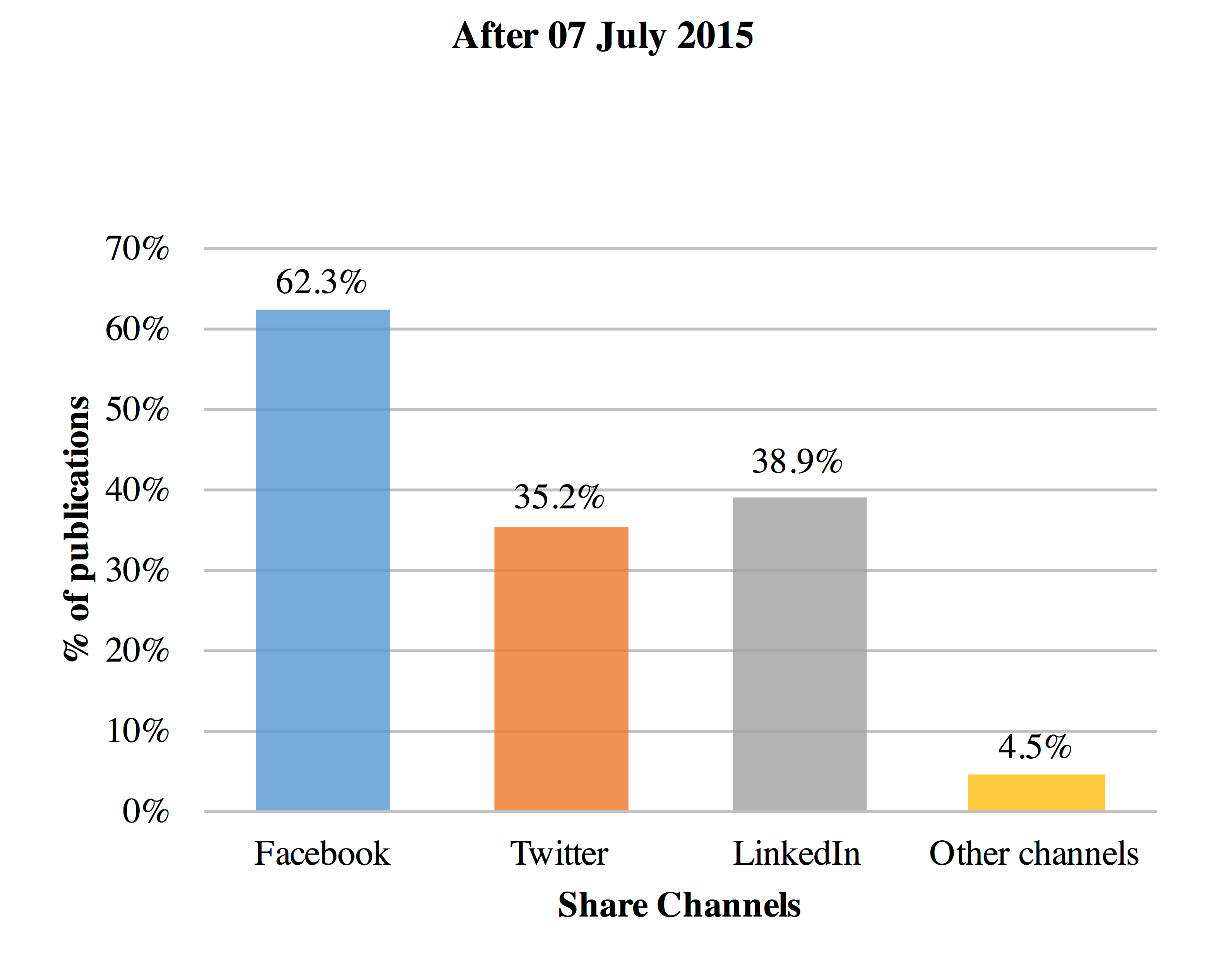

Supplement: S2 Fig — Percentages of publications on Kudos with share actions (n = 2,488) that were shared via the three social media channels: Facebook (1,551), Twitter (877), and LinkedIn (969), as well as via other channels (111), after 07 July 2015. LinkedIn was introduced by Kudos as an integrated sharing option in July 2015. Some publications were shared via multiple channels and on different share dates. Multiple shares on the same channel on the same share date were counted only once in this analysis. (TIFF) [file pone.0183217.s002.tiff]

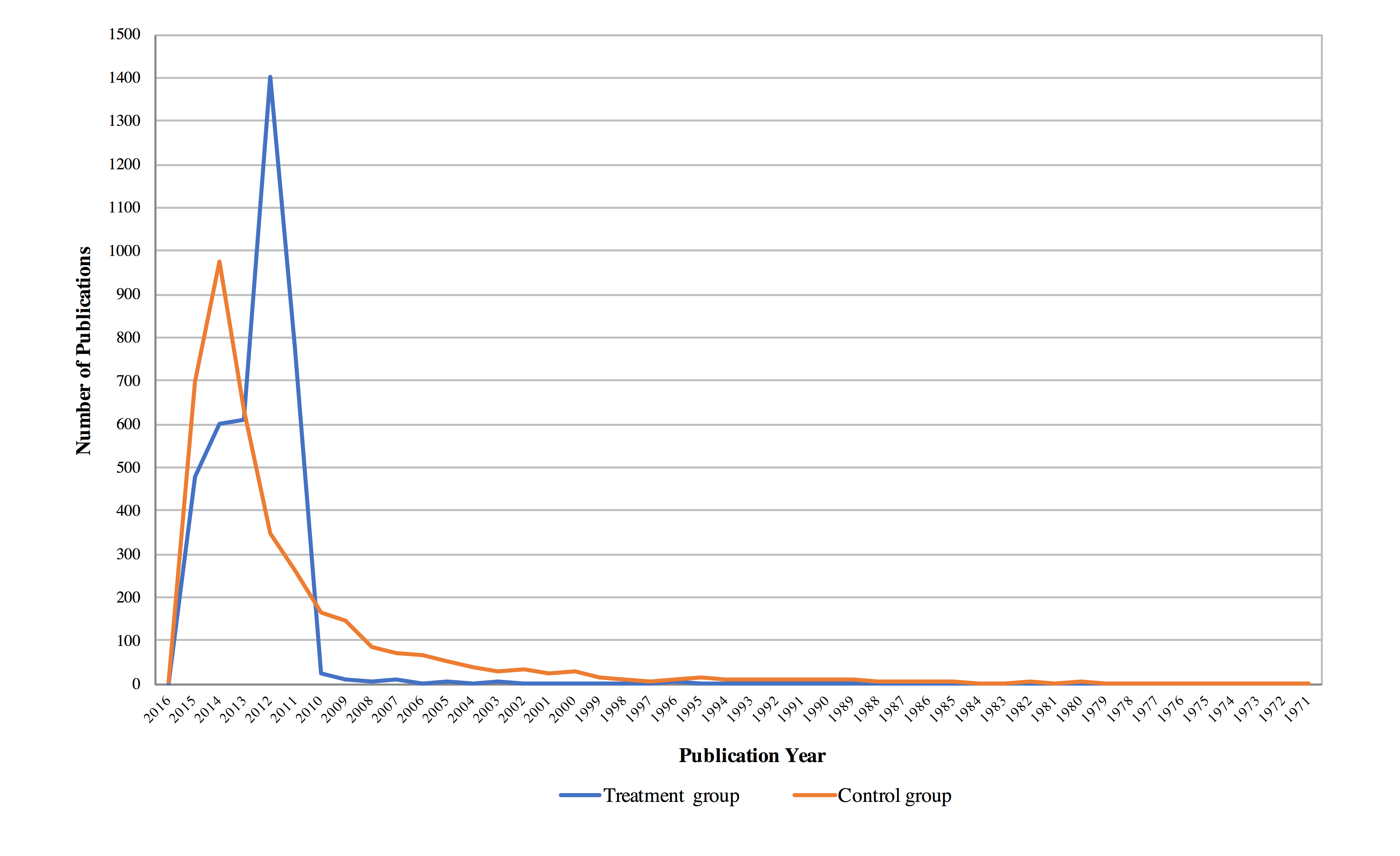

Supplement: S3 Fig — The publication years for the publications with publication dates available, and restricted to publications with document types: article, article in press, and conference paper for the Treatment group (n = 3,961) and the Control group (n = 3,851). (TIFF) [file pone.0183217.s003.tiff]

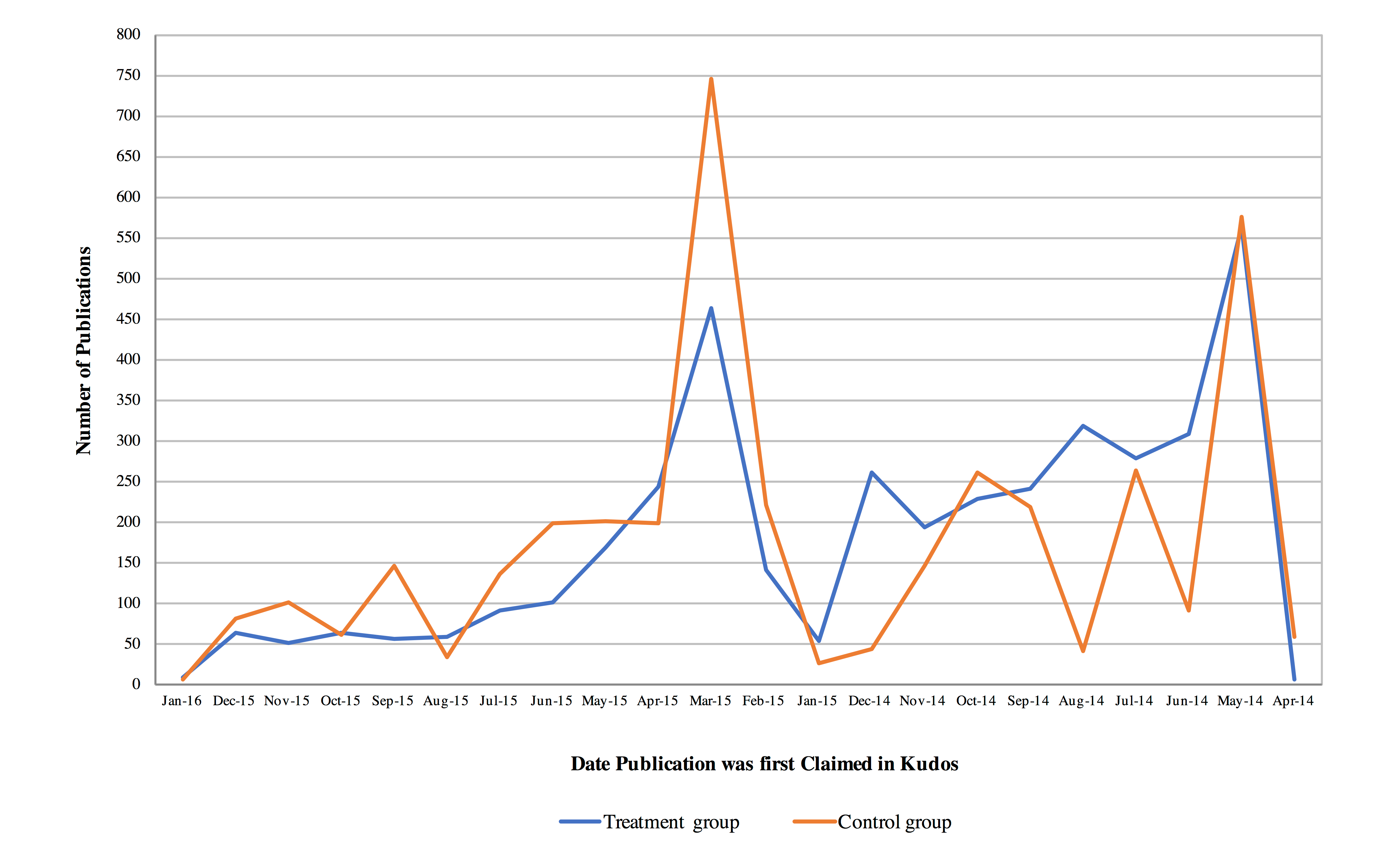

Supplement: S4 Fig — The month and year the publications were first claimed by an author in Kudos for publications with publication dates available, and restricted to publications with document types: article, article in press, and conference paper for the Treatment Group (n = 3,961) and the Control Group (n = 3,851). (TIFF) [file pone.0183217.s004.tiff]

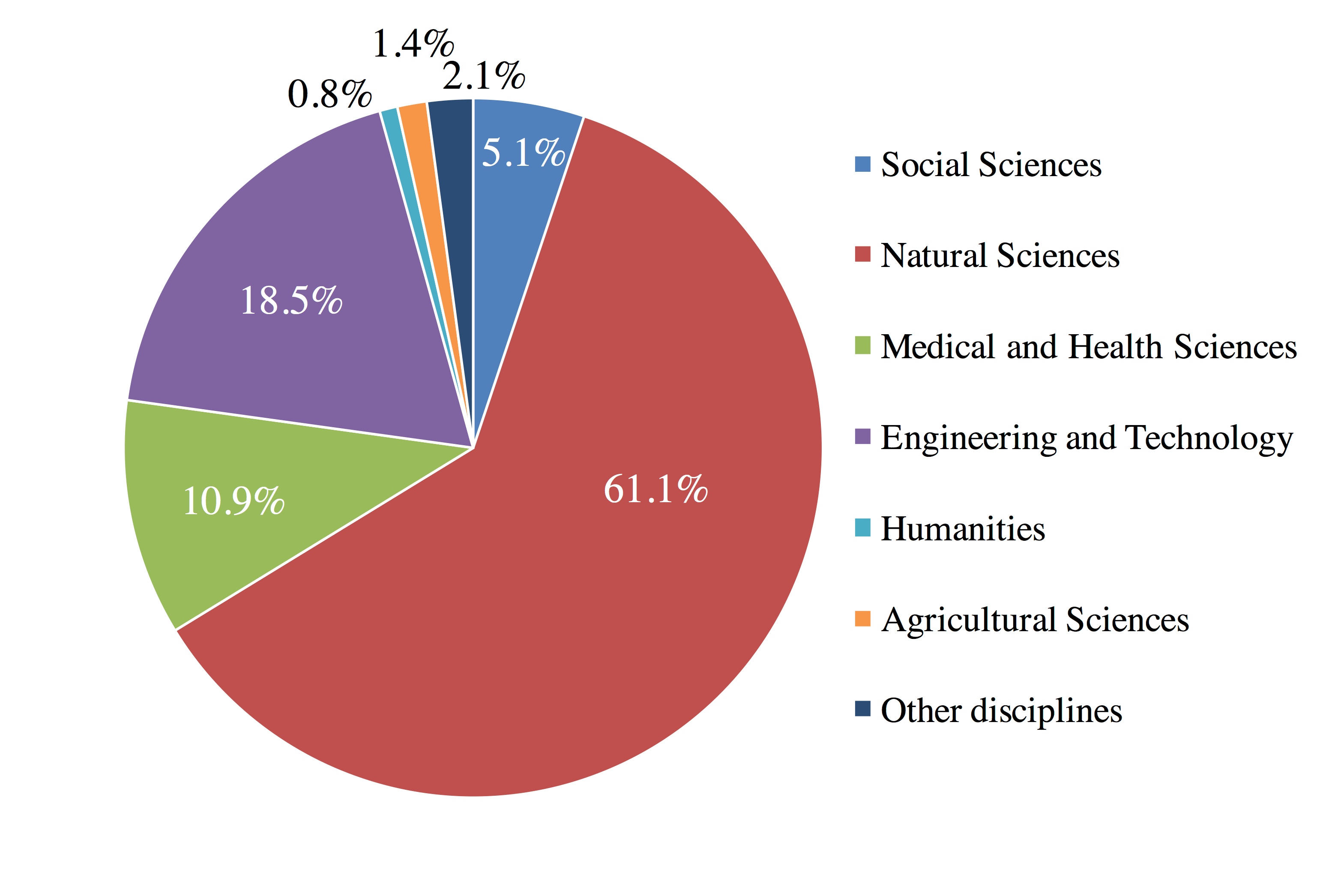

Supplement: S5 Fig — Percentage distribution of disciplines of the first authors who claimed the publications in Kudos. For publications with publication dates available and restricted to publications with document types: article, article in press, and conference paper in the Treatment Group (n = 1,692): Natural Sciences (1,034), Engineering and Technology (313), Medical and Health Sciences (185), Agricultural Sciences (23), Social Sciences (87), Humanities (14), and other disciplines (36). (TIFF) [file pone.0183217.s005.tiff]

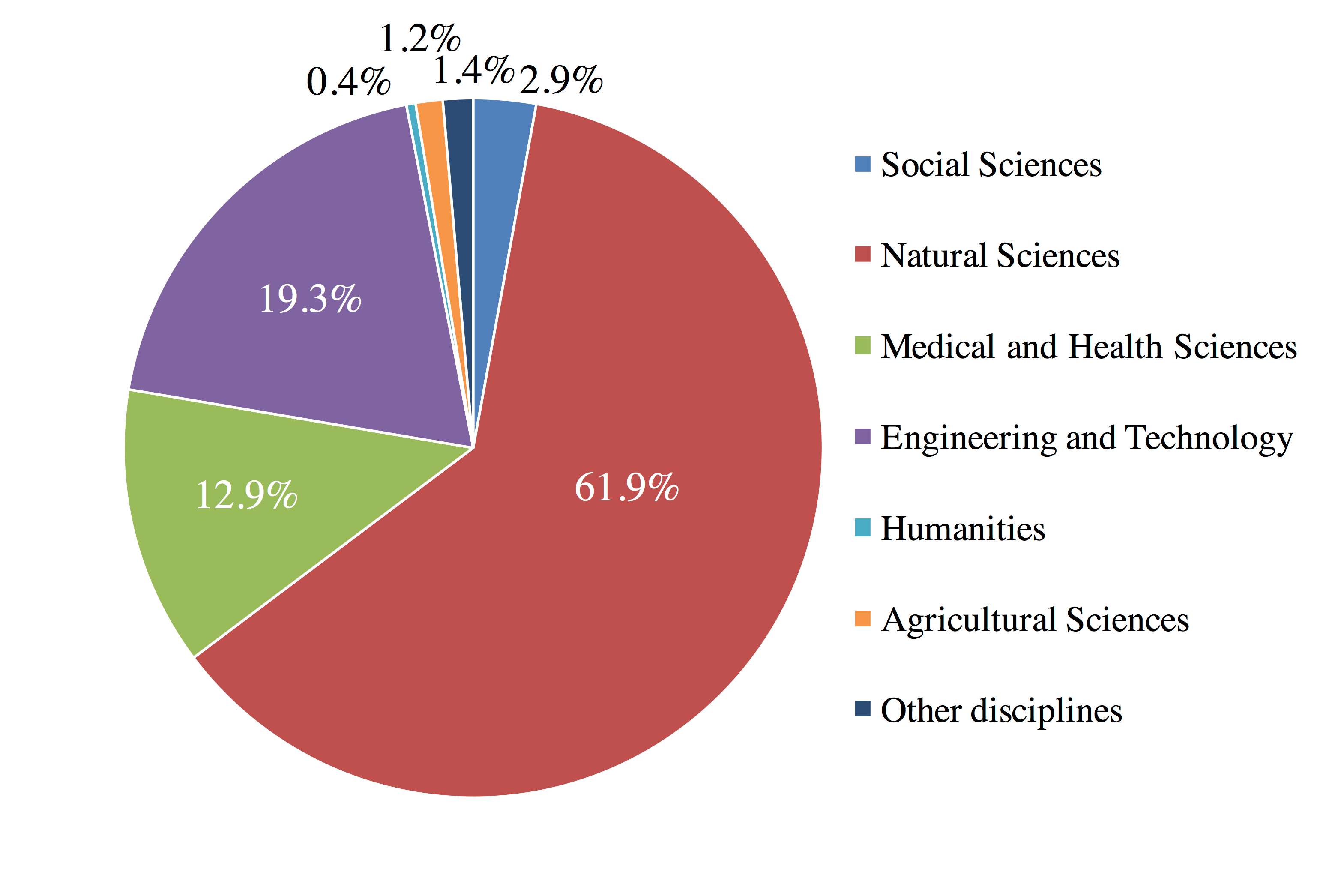

Supplement: S6 Fig — Percentage distribution of disciplines of the first authors who claimed the publications in Kudos. For publications with publication dates available and restricted to publications with document types: article, article in press, and conference paper in the Control Group (n = 3,521).: Natural Sciences (2,178), Engineering and Technology (678), Medical and Health Sciences (455), Agricultural Sciences (44), Social Sciences (102), Humanities (15), and other disciplines (49). (TIFF) [file pone.0183217.s006.tiff]
