# Supplementary material for: Analysing researchers’ outreach efforts and the association with publication metrics: A case study of Kudos
Source: PLoS One. 2017 Aug 17;12(8):e0183217. doi: 10.1371/journal.pone.0183217 (PMC5560533; doi:10.1371/journal.pone.0183217)
Supplement: S1 Table — For career levels: professionals (n = 506), students (n = 256), researchers (n = 689), faculty (n = 2,420), and other career levels (n = 241) who shared their publications via Kudos on Facebook. (PDF) [file pone.0183217.s007.pdf]

	Sharing on Facebook		
	**Yes**	**No**	***p***
**Professionals**	282 (55.7%)	224 (44.3%)	.382
**Students**	157 (61.3%)	99 (38.7%)	
**Researchers**	390 (56.6%)	299 (43.4%)	
**Faculty**	1331 (55.0%)	1089 (45.0%)	
**Other career levels**	138 (57.3%)	103 (42.7%)	
