# Supplementary material for: Analysing researchers’ outreach efforts and the association with publication metrics: A case study of Kudos
Source: PLoS One. 2017 Aug 17;12(8):e0183217. doi: 10.1371/journal.pone.0183217 (PMC5560533; doi:10.1371/journal.pone.0183217)
Supplement: S2 Table — For career levels: professionals (n = 506), students (n = 256), researchers (n = 689), faculty (n = 2,420), and other career levels (n = 241) who shared their publications via Kudos on Twitter. (PDF) [file pone.0183217.s008.pdf]

	Sharing on Twitter		
	**Yes**	**No**	***p***
**Professionals**	273 (54.0%)	233 (46.0%)	<.01
**Students**	98 (38.3%)	158 (61.7%)	
**Researchers**	335 (48.6%)	354 (51.4%)	
**Faculty**	988 (40.8%)	1432 (59.2%)	
**Other career levels**	118 (49.0%)	123 (51.0%)	
