# Supplementary material for: Analysing researchers’ outreach efforts and the association with publication metrics: A case study of Kudos
Source: PLoS One. 2017 Aug 17;12(8):e0183217. doi: 10.1371/journal.pone.0183217 (PMC5560533; doi:10.1371/journal.pone.0183217)
Supplement: S3 Table — For career levels: professionals (n = 506), students (n = 256), researchers (n = 689), faculty (n = 2,420), and other career levels (n = 241) who shared their publications via Kudos on LinkedIn. (PDF) [file pone.0183217.s009.pdf]

	Sharing on LinkedIn		
	**Yes**	**No**	***p***
**Professionals**	91 (18.0%)	415 (82.0%)	.170
**Students**	46 (18.0%)	210 (82.0%)	
**Researchers**	151 (21.9%)	538 (78.1%)	
**Faculty**	529 (21.9%)	1891 (78.1%)	
**Other career levels**	57 (23.7%)	184 (76.3%)	
