# Supplementary material for: Analysing researchers’ outreach efforts and the association with publication metrics: A case study of Kudos
Source: PLoS One. 2017 Aug 17;12(8):e0183217. doi: 10.1371/journal.pone.0183217 (PMC5560533; doi:10.1371/journal.pone.0183217)
Supplement: S4 Table — For career levels: professionals (n = 506), students (n = 256), researchers (n = 689), faculty (n = 2,420), and other career levels (n = 241) who shared their publications via Kudos on other channels. * Due to small sample sizes (n < 40), results should be interpreted with caution. (PDF) [file pone.0183217.s010.pdf]

	Sharing on Other channels		
	**Yes**	**No**	***p***
**Professionals**	112 (22.1%)	394 (77.9%)	<.01
**Students**	42 (16.4%)	214 (83.6%)	
**Researchers**	81 (11.8%)	608 (88.2%)	
**Faculty**	326 (13.5%)	2094 (86.5%)	
**Other career levels**	25* (10.4%)	216 (89.6%)	
