# Supplementary material for: Analysing researchers’ outreach efforts and the association with publication metrics: A case study of Kudos
Source: PLoS One. 2017 Aug 17;12(8):e0183217. doi: 10.1371/journal.pone.0183217 (PMC5560533; doi:10.1371/journal.pone.0183217)
Supplement: S7 Table — Authors from Social Sciences (1,147), Natural Sciences (1,045), Medical and Health Sciences (759), Engineering and Technology (590), Humanities (304), Agricultural Sciences (65), and other disciplines (102), who shared their publications via Kudos on LinkedIn. * Due to small sample sizes (n < 40), results should be interpreted with caution. (PDF) [file pone.0183217.s013.pdf]

	Sharing on LinkedIn		
	Yes	No	*p*
**Natural Sciences**	188 (18.0%)	857 (82.0%)	<.01
**Engineering and Technology**	146 (24.7%)	444 (75.3%)	
**Medical and Health Sciences**	175 (23.1%)	584 (76.9%)	
**Agricultural Sciences**	7* (10.8%)	58 (89.2%)	
**Social Sciences**	288 (25.1%)	859 (74.9%)	
**Humanities**	47 (15.5%)	257 (84.5%)	
**Other disciplines**	20* (19.6%)	82 (80.4%)	
