# Supplementary material for: Analysing researchers’ outreach efforts and the association with publication metrics: A case study of Kudos
Source: PLoS One. 2017 Aug 17;12(8):e0183217. doi: 10.1371/journal.pone.0183217 (PMC5560533; doi:10.1371/journal.pone.0183217)
Supplement: S9 Table — The career levels of the first authors who claimed publications on Kudos were re-coded using the five broad OECD (Organisation for Economic Co-operation and Development, https://www.oecd.org/science/inno/38235147.pdf) career categories: Professionals, students, researchers, faculty, and other career levels. (PDF) [file pone.0183217.s015.pdf]

**Professionals**<br><br>• Association or Advocacy group<br>• Consultant<br>• Business/ Corporate Professional<br>• Government Employee<br>• Health Care Professional<br>• Media Professional	**Students**<br><br>• Graduate Student<br>• Undergraduate
**Researchers**<br><br>• Post-Doc<br>• Research Associate<br>• Research Fellow	**Faculty**<br><br>• Emeritus<br>• Faculty Member<br>• Lecturer<br>• Professor<br>• Reader
**Other career levels**<br><br>• Other	
