# Supplementary material for: Analysing researchers’ outreach efforts and the association with publication metrics: A case study of Kudos
Source: PLoS One. 2017 Aug 17;12(8):e0183217. doi: 10.1371/journal.pone.0183217 (PMC5560533; doi:10.1371/journal.pone.0183217)
Supplement: S10 Table — The disciplines of the first authors who claimed publications in Kudos were recoded using the OECD (Organisation for Economic Co-operation and Development, https://www.oecd.org/science/inno/38235147.pdf) classification scheme, which provides seven broad disciplines: Natural Sciences, Engineering and Technology, Medical and Health Sciences, Agricultural Sciences, Social Sciences, Humanities, and other disciplines. (PDF) [file pone.0183217.s016.pdf]

**Natural Sciences**<br><br>• Statistics<br>• Physics<br>• Mathematics<br>• Environmental Sciences<br>• Earth and Planetary Sciences<br>• Computer Science<br>• Chemistry<br>• Geography<br>• Aquatic Sciences<br>• Life Sciences	**Engineering and Technology**<br><br>• Materials Science<br>• General Science & Engineering<br>• Food Science & Technology<br>• Engineering & Technology<br>• Civil Engineering & Construction<br>• Chemical Engineering<br>• Energy
**Medical and Health Sciences**<br><br>• Nursing, Dentistry & Healthcare<br>• Medicine And Medical Sciences<br>• Health Sciences<br>• Dentistry	**Agricultural Sciences**<br><br>• Veterinary Medicine<br>• Agriculture Sciences
**Social Sciences**<br><br>• Urban Studies<br>• Tourism, Hospitality & Events<br>• Sports & Leisure<br>• Sociology<br>• Political Science<br>• Library and Information Science<br>• Law & Criminology<br>• Finance<br>• Family & Child Studies<br>• Education<br>• Economics<br>• Communication & Media Studies<br>• Business & Management<br>• Asian Studies<br>• Area Studies<br>• Anthropology<br>• Psychology<br>• Social Policy & Welfare	**Humanities**<br><br>• Religion & Theology<br>• Philosophy<br>• Literature<br>• Language & Linguistics<br>• History<br>• Art & Applied Arts<br>• Architecture & Planning<br>• Archaeology<br>• Foreign Languages<br>• European Studies<br>• Development Studies<br>• Cultural Studies<br>• Classical Studies<br>• African Studies
**Other disciplines**<br><br>• Other<br>• Innovation	
