# Supplementary material for: Analysing researchers’ outreach efforts and the association with publication metrics: A case study of Kudos
Source: PLoS One. 2017 Aug 17;12(8):e0183217. doi: 10.1371/journal.pone.0183217 (PMC5560533; doi:10.1371/journal.pone.0183217)
Supplement: S11 Table — The document type was retrieved from Scopus for the publications in the Treatment group (n = 4,867) and the Control group (n = 4,867). Only 4,208 had document types available in the Treatment group, and only 4,155 had document types available in the Control group. The document types: article, article in press, conference paper, editorial, erratum, letter, note, review, and short survey were available. (PDF) [file pone.0183217.s017.pdf]

	Treatment group	Control group
**Article**	3938	3801
**Article in Press**	9	2
**Conference Paper**	14	48
**Editorial**	14	18
**Erratum**	3	20
**Letter**	3	17
**Note**	10	23
**Review**	216	220
**Short Survey**	1	6
**Not Available**	659	712
**Total**	4,867	4,867
