# Supplementary material for: Analysing researchers’ outreach efforts and the association with publication metrics: A case study of Kudos
Source: PLoS One. 2017 Aug 17;12(8):e0183217. doi: 10.1371/journal.pone.0183217 (PMC5560533; doi:10.1371/journal.pone.0183217)
Supplement: S12 Table — Full text downloads for publications on the day they were first claimed by an author in Kudos, on the extraction date from Kudos for this study (30 January 2016), and the calculated increase in full text downloads between the claim date and extraction date, for publications with publication dates available and restricted to publications with document types: article, article in press, and conference paper for the Treatment group (n = 3,961) and the Control group (n = 3,851). (PDF) [file pone.0183217.s018.pdf]

	Metric on claim date		Metric on extraction date		Increase in Metric	
	**Treatment**	**Control**	**Treatment**	**Control group**	**Treatment**	**Control**
**Minimum**	0.00	0.00	0.00	0.00	0.00	0.00
**1st Quartile**	124.00	2.00	391.00	83.00	80.00	51.00
**Median**	347.00	43.00	532.00	240.00	158.00	128.00
**Mean**	378.37	205.66	644.58	454.32	266.21	248.66
**3rd Quartile**	486.00	260.00	716.00	611.00	282.00	299.50
**Maximum**	30477	12942	31419	13586	18520	8290
**Standard Deviation**	881.06	476.47	1067.07	684.50	551.74	393.47
**Skew**	26.83	11.83	17.91	6.85	15.27	6.37
**Kurtosis**	852.43	252.62	434.93	89.97	382.88	77.34
